# Supplementary material for: Formyl-peptide receptor type 2 activation mitigates heart and lung damage in inflammatory arthritis
Source: EMBO Mol Med. 2025 Apr 3;17(5):1153–83. doi: 10.1038/s44321-025-00227-1 (PMC12081931; doi:10.1038/s44321-025-00227-1)
Supplement: Supplementary file 1 — Table EV1 [file 44321_2025_227_MOESM1_ESM.docx]

**Table EV1: Plasma cytokines, chemokines, and other mediators (Data are in pg/mL).**

| **Marker** | **Naïve control** | **HH** | **STIA** | **HH+STIA (Vehicle)** | **HH+STIA**  **(BMS235)** | **HH+STIA (C43)** |
| --- | --- | --- | --- | --- | --- | --- |
| **Adiponectin** | 878325  ± 119872 | 650000  ± 67860 | 690299  ± 73838 | 851258  ± 125348 | 894522  ± 150237 | 875891  ± 140115 |
| **C Reactive**  **Protein** | 5517980 ±  1038018 | 15634987± 1958468 | 11458850 ±  2257080 | 8762784 ±  1334388 | 14530297  ± 6314870 | 10618207  ± 1403711 |
| **Cystatin C** | 156063  ± 56935 | 273974  ± 98537 | 226437  ± 87321 | 152438  ± 55901 | 252757  ± 86993 | 141939  ± 38609 |
| **Endoglin/CD105** | 4291  ± 430 | 5393  ± 721 | 4629  ± 419 | 4469  ± 404 | 3885  ± 410 | 5215  ± 535 |
| **Endostatin** | 12018  ± 1622 | 17596  ± 3174 | 11061  ± 884 | 13531  ± 3176 | 17068  ± 2567 | 14853  ±b2030 |
| **FABP4/ A-FABP** | 3065  ± 0 | 3065  ± 0 | 5517  ± 2452 | 3656  ± 592 | 3451  ± 386 | 3065  ± 0 |
| **Fas (APO-1)** | 84.9  ± 11 | 75.5  ± 5.0 | 102  ± 18 | 93.2  ± 9.8 | 73.2  ± 8.7 | 89.9  ± 8.4 |
| **FGF-21** | 67.9  ± 15.3 | 66.4  ± 10.9 | 36.3  ± 4.4 | 69.6  ± 14.9 | 65.6  ± 11.1 | 46.3  ± 3.4 |
| **FGF-23** | 154  ± 15.4 | 144  ± 9.8 | 202  ± 56.7 | 178  ± 5.4 | 134  ± 12.8 | 166  ± 15.9 |
| **Galectin-3** | 1141  ± 101 | 1352  ± 247 | 1477  ± 198 | 1248  ± 208 | 1248  ± 208 | 1542  ± 233 |
| **GDF-15** | 7.22  ± 0.5 | 4.72  ± 0.2 | 3.97  ± 0.1 ***** | 5.78  ± 0.8 | 6.00  ± 0.7 | 6.41  ± 0.5 |
| **IL-1β** | 11.0  ± 3.4 | 10.0  ± 0.63 | 24.2  ± 7.0 | 10.7  ± 2.5 | 11.8  ± 3.2 | 15.7  ± 3.1 |
| **IL-10** | 2.60  ± 0.1 | 34.1  ± 31.2 | 130  ± 127 | 2.83  ± 0.1 | 27.7  ± 25.0 | 27.9  ± 24.9 |
| **IL-6** | 9.19  ± 1.6 | 12.7  ± 2.6 | 19.2  ± 5.5 | 12.7  ± 1.6 | 10.6  ± 2.0 | 17.6  ± 1.9 |
| **Lipocalin-2/NGAL** | 33350  ± 1187 | 52389  ± 19243 | 70612  ± 8149 | 52614  ± 10384 | 42179  ± 4153 | 110440  ± 45480 |
| **MMP-12** | 44.9  ± 7.8 | 34.1  ± 2.4 | 143  ± 96.7 | 36.7  ± 5.1 | 49.6  ± 17.3 | 54.7  ± 8.0 |
| **MMP-2** | 1149989  ± 4608 | 102687  ± 22916 | 98918  ± 4573 | 106608  ± 14598 | 124890  ± 16116 | 83325  ± 5435 |
| **MMP-3** | 3780  ± 0 | 3308  ± 472 | 4914  ± 1134 | 3780  ± 0 | 3024  ± 463 | 3780  ± 0 |
| **MMP-8** | 11491  ± 1788 | 17708  ± 4137 | 32993  ± 5039 ***** | 21783  ± 4156 | 11130  ± 2366 | 21703  ± 4914 |
| **MMP-9** | 6874  ± 341 | 8225  ± 1318 | 11371  ± 2289 | 7731  ± 814 | 5995  ± 846 | 9251  ± 1932 |
| **MPO** | 11398  ± 1110 | 13815  ± 2361 | 22285  ± 4534 | 19562  ± 1953 | 14705  ± 1744 | 14884  ± 1864 |
| **Osteoprotegerin** | 666  ± 26 | 641  ± 55 | 700  ± 82 | 691  ± 71 | 629  ± 89 | 718  ± 83 |
| **Osteopontin** | 32622  ± 5595 | 21389  ± 510 | 26803  ± 5363 | 25871  ± 2766 | 24949  ± 3509 | 21496  ± 565 |
| **PAI-1/Serpin E1** | 315  ± 18 | 556  ± 65 | 832  ± 107 ***** | 683  ± 40 | 477  ± 45 | 710  ± 106 |
| **Pentraxin-3** | 6022  ± 457 | 7370  ± 1459 | 9229  ± 2318 | 5245  ± 952 | 6674  ± 749 | 10022  ± 1888 |
| **Renin** | 22213  ± 1757 | 24116  ± 4073 | 22529  ± 3571 | 28849  ± 4073 | 22953  ± 4382 | 29504  ± 4909 |
| **sFlt-1** | 1522  ± 202 | 3225  ± 1151 | 3586  ± 1568 | 2106  ± 260 | 1428  ± 151 | 2057  ± 159 |
| **Soluble ICAM-1** | 5224  ± 1760 | 10753  ± 1107 | 9720  ± 1572 | 10640  ± 904 | 8551  ± 1010 | 11153  ± 1041 |
| **Soluble P-Selectin** | 64323  ± 810 | 38076  ± 1604 | 60336  ± 8817 | 46488  ± 4730 | 52602  ± 8149 | 50258  ± 7585 |
| **Soluble ST-2** | 19639  ± 1373 | 24148  ± 4893 | 27350  ± 1283 | 26728  ± 2210 | 19543  ± 1241 | 26226  ± 2514 |
| **Soluble TNF RI** | 770  ± 0 | 770  ± 0 | 1301  ± 531 | 927  ± 157 | 770 ±  0 | 770 ±  0 |
| **Soluble TNF RII** | 421  ± 18 | 422  ± 89 | 488  ± 70 | 472  ± 70 | 553  ± 65 | 535  ± 75 |
| **Syndecan-1** | 3341  ± 112 | 3158  ± 709 | 5802  ± 1811 | 3497  ± 342 | 2635  ± 855 | 3959  ± 163 |
| **Tenascin C** | 11664  ± 269 | 16451  ± 2947 | 14632  ± 1040 | 14887  ± 2348 | 17127  ± 2019 | 13725  ± 2284 |
| **TIM-1/KIM-1** | 14.5  ± 4.4 | 19.7  ± 2.2 | 20.7  ± 9.2 | 11.6  ± 2.9 | 15.1  ± 3.4 | 11.9  ±v3.0 |
| **TIMP-1** | 1563  ± 57 | 2081  ± 391 | 2782  ± 503 | 2223  ± 238 | 1875  ± 193 | 2359  ± 353 |
| **TIMP-4** | 127  ± 29.1 | 100  ± 10.3 | 94  ± 9.4 | 111  ± 12.2 | 123  ± 7.1 | 121  ± 6.2 |
| **TNF-α** | 0.55  ± 0.02 | 0.78  ± 0.06 | 1.36  ± 0.74 | 0.53  ± 0.04 | 0.65  ± 0.06 | 0.68  ± 0.06 |
| **VEGF-A** | 7.88  ± 0.75 | 9.82  ± 1.3 | 11.8  ± 3.3 | 9.91  ± 0.88 | 7.47  ± 0.80 | 9.85  ± 0.83 |
| **BNP** | 0.30  ± 0 | 0.67  ± 0.4 | 1.01  ± 0.4 | 0.40  ± 0.06 | 0.48  ± 0.18 | 0.30  ± 0 |
| **CCL21/Exodus-2** | 1332  ± 155 | 1830  ± 179 | 1933  ± 163 | 1758  ± 132 | 1555  ± 123 | 1779  ± 168 |
| **CCL11/Eotaxin** | 1497  ± 360 | 1561  ± 251 | 1556  ± 119 | 1359  ± 179 | 1282  ± 143 | 1579  ± 143 |
| **Erythropoietin** | 419  ± 62 | 312  ± 104 | 763  ± 568 | 236  ± 64 | 422  ± 76 | 300  ± 31 |
| **CX3CL1/**  **Fractalkine** | 237  ± 15 | 252  ± 15 | 315  ± 105 | 225  ± 14 | 223  ± 16 | 241  ± 10 |
| **G-CSF** | 318  ± 35 | 362  ± 102 | 629  ± 114 | 302  ± 67 | 576  ± 161 | 490  ± 81 |
| **GM-CSF** | 14.7  ± 0 | 18.3  ± 3.7 | 67.5  ± 29 | 17.6  ± 2.9 | 17.6  ± 2.9 | 14.6  ± 0 |
| **CXCL1/KC** | 213  ± 36 | 161  ± 74 | 267  ± 116 | 578  ± 409 | 265  ± 49 | 413  ± 100 |
| **CXCL2 /MIP-2α ** | 74.5  ± 10.7 | 92.0  ± 19.7 | 128  ± 56.8 | 76.9  ± 11.1 | 70.7  ± 13.3 | 82.6  ± 24.8 |
| **IFNβ-1** | 220  ± 73 | 419  ± 285 | 427  ± 263 | 163  ± 53 | 202  ± 56 | 201  ± 56 |
| **IFN-γ** | 1.64  ± 0 | 2.05  ± 0.4 | 4.92  ± 2.9 | 2.30  ± 0.4 | 1.64  ± 0 | 1.97 ±  0.330 |
| **IL-1α** | 22.4  ± 4.7 | 110  ± 84 | 44.7  ± 22 | 19.9  ± 2.9 | 39.8  ± 19 | 31.7  ± 10 |
| **IL-11** | 36.6  ± 0 | 59.3  ± 22.7 | 65.9  ± 29.0 | 36.6  ± 0 | 36.6  ± 0 | 36.6  ± 0 |
| **IL-12 (p40)** | 29.3  ± 0 | 25.6  ± 3.7 | 52.7  ± 23.4 | 29.3  ± 0 | 29.3  ± 0 | 29.3  ± 0 |
| **IL-12 (p70)** | 1.58  ± 0 | 2.36  ± 0.45 | 2.84  ± 1.26 | 1.58  ± 0 | 2.44  ± 0.56 | 1.89  ± 0.32 |
| **IL-13** | 117  ± 0 | 117  ± 0 | 211  ± 94 | 117  ± 0 | 117  ± 0 | 117  ± 0 |
| **IL-15** | 91.7  ± 71.2 | 272  ± 38.1 | 132  ± 26.6 | 67.1  ± 41.9 | 148  ± 52.6 | 68.0  ± 20.8 |
| **IL-16** | 663  ± 44 | 3468  ± 2235 | 4716  ± 1535 | 3171  ± 805 | 1611  ± 467 | 4645  ± 1804 |
| **IL-17A/CTLA8** | 2.46  ± 1.32 | 2.46  ± 1.69 | 1.73  ± 0.45 | 0.79  ± 0.13 | 0.92  ± 0.16 | 1.31  ± 0.51 |
| **IL-17A/F** | 73  ± 0 | 192  ± 99 | 271  ± 179 | 117  ± 19 | 100  ± 16 | 166  ± 66 |
| **IL-17E/IL-25** | 832  ± 172 | 1000  ± 79 | 1084  ± 502 | 865  ± 132 | 941  ± 141 | 1199  ± 181 |
| **IL-17F** | 21.6  ± 3.8 | 95.8  ± 79.2 | 47.2  ± 29.3 | 18.1  ± 3.1 | 18.3  ± 3.5 | 29.1  ± 15.2 |
| **IL-2** | 1.31  ± 0 | 2.57  ± 1.3 | 2.36  ± 1.0 | 11.31  ± 0 | 1.31  ± 0 | 1.31  ± 0 |
| **IL-20** | 88  ± 34 | 245  ± 112 | 210  ± 36 | 224  ± 110 | 213  ± 72 | 136  ± 43 |
| **IL-21** | 255  ± 104 | 1242  ± 982 | 472  ± 202 | 391  ± 109 | 185  ± 20.5 | 738  ± 466 |
| **IL-22** | 41.4  ± 21.7 | 210.8  ± 82.2 * | 52.7  ± 12.4 | 54.1  ± 8.8 | 22.0  ± 3.8 | 42.8  ± 10.5 |
| **IL-23** | 911  ± 227 | 788  ± 196 | 1066  ± 250 | 820  ± 137 | 643  ± 41 | 820  ± 137 |
| **IL-27** | 1758  ± 0 | 2210  ± 452 | 3164  ± 1406 | 1758  ± 0 | 1608  ± 150 | 1757  ± 0 |
| **IL-28B/IFNL3** | 254  ± 0 | 254  ± 0 | 330  ± 76 | 254  ± 0 | 254  ± 0 | 254  ± 0 |
| **IL-3** | 29.3  ± 0 | 29.3  ± 0 | 52.7  ± 23 | 29.3  ± 0 | 29.3  ± 0 | 29.3  ± 0 |
| **IL-31** | 130  ± 32 | 86.5  ± 6.4 | 273  ± 176 | 117  ± 19 | 117  ± 19 | 97.7  ± 0 |
| **IL-33** | 78.1  ± 0 | 179  ± 100 | 141  ± 62 | 78.1  ± 0 | 78.1  ± 0 | 78.1  ± 0 |
| **IL-4** | 0.13  ± 0 | 0.13  ± 0 | 0.23  ± 0.1 | 0.13  ± 0 | 0.13  ± 0 | 0.13  ± 0 |
| **IL-5** | 1.31  ± 0 | 1.31  ± 0 | 2.36  ± 1.1 | 1.31  ± 0 | 1.31  ± 0 | 1.31  ± 0 |
| **IL-7** | 2.7  ± 0.5 | 26.8  ± 23.5 | 5.9  ± 2.6 | 2.9  ± 0.3 | 3.3  ± 0 | 2.6  ± 0.4 |
| **IL-9** | 174  ± 60 | 156  ± 47 | 296  ± 137 | 125  ± 31 | 115  ± 33 | 113  ± 34 |
| **CXCL10/IP-10** | 65.1  ± 9.4 | 76.7  ± 5.0 | 103  ± 25 | 77.5  ± 12.3 | 71.5  ± 5.8 | 65.6  ± 4.5 |
| **LIF** | 14.6  ± 0 | 13.8  ± 0.8 | 26.4  ± 11.7 | 14.6  ± 0 | 14.6  ± 0 | 16.8  ± 3.2 |
| **CXCL6/LIX** | 508  ± 233 | 1513  ± 892 | 532  ± 241 | 209  ± 56 | 500  ± 151 | 261  ± 71 |
| **CCL2/MCP-1/JE** | 5.25  ± 0 | 6.56  ± 1.3 | 14.5  ± 5.7 | 7.82  ± 1.6 | 5.25  ± 0 | 11.4  ± 6.2 |
| **MCP-5** | 107  ± 23 | 108  ± 32 | 160  ± 35 | 144  ± 28 | 134  ± 10 | 136  ± 22 |
| **M-CSF** | 19.5  ± 5.0 | 24.0  ± 5.3 | 46.9  ± 25.1 | 22.1  ± 4.5 | 22.2  ± 4.4 | 23.4  ± 3.6 |
| **CCL22/MDC** | 121  ± 24 | 195  ± 16 | 296  ± 63 | 203  ± 31 | 165  ± 18 | 210  ± 38 |
| **CXCL9/MIG** | 396  ± 71 | 490  ± 153 | 326  ± 81 | 548  ± 128 | 342  ± 38 | 341  ± 47 |
| **CCl3/MIP-1α ** | 58.4  ± 29.3 | 62.2  ± 19.7 | 83.3  ± 22.1 | 64.0  ± 21.9 | 66.1  ± 13.3 | 58.7  ± 15.1 |
| **CCL4/MIP-1β ** | 27.1  ± 6.2 | 31.0  ± 2.2 | 65.6  ± 31.2 | 28.3  ± 2.9 | 50.0  ± 21.2 | 28.8  ± 4.2 |
| **CCL20/MIP-3α** | 58.0  ± 20.7 | 43.2  ± 5.8 | 195  ± 73.3 | 67.4  ± 24.3 | 59.6  ± 20.1 | 46.0  ± 13.3 |
| **CCL19/MIP-3β** | 73.2  ± 0 | 68.1  ± 5.1 | 132  ± 58.6 | 73.2  ± 0 | 73.2  ± 0 | 73.2  ± 0 |
| **CCL5/RANTES** | 24.3  ± 3.9 | 22.7  ± 3.8 | 36.0  ± 13.3 | 22.9  ± 4.0 | 22.8  ± 2.7 | 22.7  ± 2.7 |
| **sCD40L** | 97.7  ± 0 | 327  ± 229 | 186  ± 79 | 59.8  ± 9.5 | 87.9  ± 9.8 | 133  ± 35 |
| **CCL17/TARC** | 12.9  ± 1.0 | 26.4  ± 6.8 | 32.3  ± 10.3 | 17.3  ± 3.4 | 13.9  ± 1.3 | 20.8  ± 2.7 |
| **TNF-α** | 4835  ± 1762 | 11893  ± 7085 | 3506  ± 671 | 4331  ± 972 | 3460  ± 413 | 3988  ± 278 |

ANOVA for all groups from one single experiment; post-hoc Tukey test for significance were performed for *vs.* naïve or *vs.* HH+STIA vehicle as appropriate according to experimental protocol. * p<0.05 vs. naive control.

Data are mean ± SEM of n=3 (naïve), n=4 (HH) and n=5 (STIA).

Data are mean ± SEM of n=5 mice per group (vehicle, BMS235 and C43 – all HH+STIA mice).
