# Supplementary material for: Formyl-peptide receptor type 2 activation mitigates heart and lung damage in inflammatory arthritis
Source: EMBO Mol Med. 2025 Apr 3;17(5):1153–83. doi: 10.1038/s44321-025-00227-1 (PMC12081931; doi:10.1038/s44321-025-00227-1)
Supplement: Supplementary file 2 — Appendix [file 44321_2025_227_MOESM2_ESM.pdf]

Appendix

Formyl-peptide receptor type 2 activation mitigates secondary organ damage in inflammatory arthritis.

Andreas Margraf, Jianmin Chen, Marilena Christoforou, Pol Claria-Ribas, Ayda Henriques Schneider, Chiara Cecconello, Weifeng Bu, Paul RC Imbert, Thomas D Wright, Stefan Russo, Isobel A Blacksell, Duco S Koenis, Jesmond Dalli, John A Lupisella, Nicholas R Wurtz, Ricardo A Garcia, Dianne Cooper, Lucy V Norling, Mauro Perretti

**Table of Content**	**Page**
Appendix Table S1<br>Haematological Parameters	**2**
Appendix Fig. S1.<br>Blood pressure and heart gating strategy	**3**
Appendix Fig. S2.<br>Heart cell characterisation through flow cytometry analyses of heart digests	**4**
Appendix Fig. S3.<br>Lung cell characterisation through flow cytometry analyses of lung digests	**5**
Appendix Fig. S4.<br>Flow cytometry gating strategy for lung cells	**6**
Appendix Fig. S5.<br>Schematic of the pharmacological study and additional echocardiography parameters	**7**
Appendix Fig. S6.<br>Individual channels for immunofluorescence of the heart	**8**
Appendix Fig. S7.<br>Individual channels for immunofluorescence of the lung	**9**
Appendix Fig. S8.<br>Individual Western blots for human monocytes	**10**
Appendix Fig. S9.<br>Flow cytometry gating strategy for paw cells	**11**

Appendix Table S1. Haematological Parameters

Parameter	Naïve	STIA	HH	HH+STIA
Red blood cells [no. x10^6/µL]	9.6±-0.3	9.7±0.4	9.3±0.2	9.5±0.1
HGB [g/dL]	13.7±0.11	13.4±0.49	13.0±0.29	13.1±0.21
Haematocrit [%]	44.7±0.58	43.7±1.95	42.92±1.06	42.9±0.79
Reticulocytes [no. x10^3/µL]	259±128	408±26	351±20	404±28
Platelets [no. x10^3/µL]	977±78.6	1090±45.7	924±58.7	1201±21.1*
PDW [fL]	6.57±0.14	6.16±0.09*	6.97±0.13	6.22±0.09*
MPV [fL]	8.27±0.07*	7.96±0.07*	9.0±0.07	7.94±0.05*
P-LCR [%]	1.97±0.88	0.98±0.26*	2.40±0.50	1.08±0.22*
PCT [%]	0.81±0.07	0.87±0.04	0.83±0.04	0.94±0.02
White blood cells [no. x10^3/µL]	6.23±0.39	4.59±0.46	3.67±0.14 #	3.03±0.20 #
Neutrophils [no. x10^3/µL]	0.71±0.04	0.51±0.07	0.35±0.034	0.48±0.06
Lymphocytes [no. x10^3/µL]	5.21±0.34	3.80±0.36	3.17±0.17 #	2.43±0.19 #
Monocytes [no. x10^3/µL]	0.14±0.01	0.20±0.08	0.08±0.003	0.07±0.01
Eosinophils [no. x10^3/µL]	0.16±0.01	0.07±0.01 #	0.06±0.01 #	0.04±0.004 #
Basophils [no. x10^3/µL]	0.003±0.003	0.002±0.002	0.003±0.003	0.004±0.002
Neutrophils [%]	11.5±0.14	11.2±1.32	9.7±1.26	16.1±2.06
Lymphocytes [%]	83.5±0.30	83.0±1.70	86.3±1.45	80.0±2.04
Monocytes [%]	2.33±0.18	4.04±1.23	2.12±0.14	2.40±0.202
Eosinophils [%]	2.63±0.12	1.68±0.14	1.725±0.21	1.32±0.12 #
Basophils [%]	0.03±0.03	0.04±0.04	0.07±0.07	0.14±0.09
RET-He [pg]	16.8±0.26	16.2±0.36	16.4±0.13	16.5±0.10
RBC-He [pg]	15.5±0.23	14.9±0.11 #	15.2±0.04	15.0±0.04
MCV [fL]	46.7±0.81	44.9±0.52	46.0±0.141	45.2±0.24
Plasma Creatinine [mg/dL]	0.34±0.13	0.97±0.26	0.49±0.09	0.49±0.07
Plasma AST [ng/mL]	188±9.0	222±5.4*#	163±1.7	196±9.4*

Values are presented as mean ± SEM of n=3 (Naïve) or n=4 (HH) or n=5 (STIA and HH+STIA) mice. Abbreviations: HGB: Haemoglobin; PDW: Platelet distribution width; MPV: Mean platelet volume; P-LCR: Platelet large cell ratio; PCT: Platelet crit; RET-He: Reticulocyte hemoglobin equivalent; RBC-He: Red blood cell hemoglobin equivalent; MCV: Mean corpuscular volume. Asterisk indicates significant differences which are detailed below.

* Significance vs. HH
\# Significance vs. Naïve
with $p \leq 0.05$ for indicated groups.

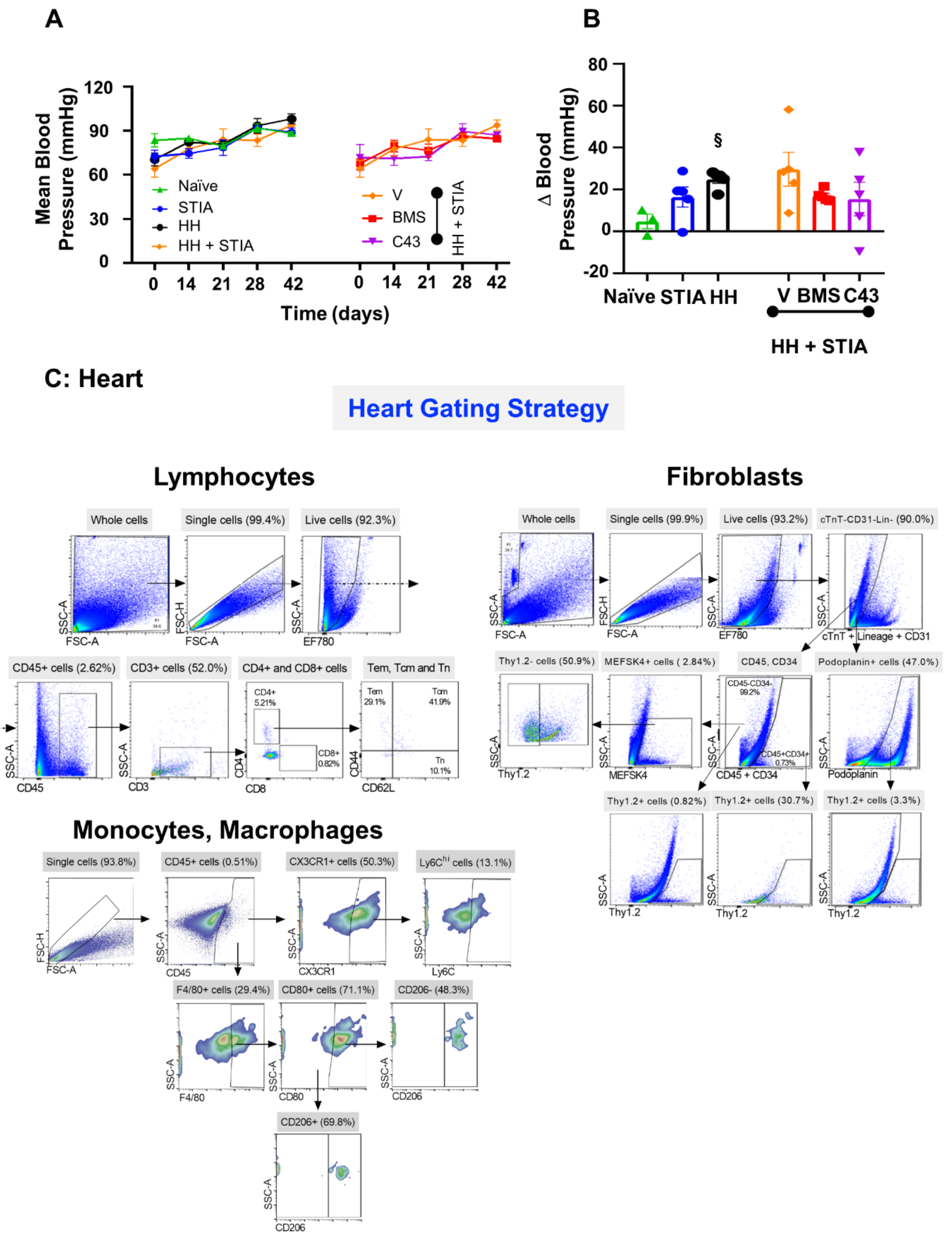

Appendix Fig. S1. Blood pressure and heart gating strategy. (A) Blood pressure over the full time course (see Figure 1). (B) Changes in blood pressure from day 0 to day 42. Data are mean±SEM of n=3 (naïve), n=4 (HH), n=5 (STIA, HH+STIA) and n=5 for vehicle (V), BMS and C43 mice. §p<0.05 *vs.* naïve mice. (C) Flow cytometry gating strategy for heart cells (N.B. the dot blot for CD45$^-$CD4$^-$Thy1.2$^+$ fibroblasts is re-used from Fig 5C).

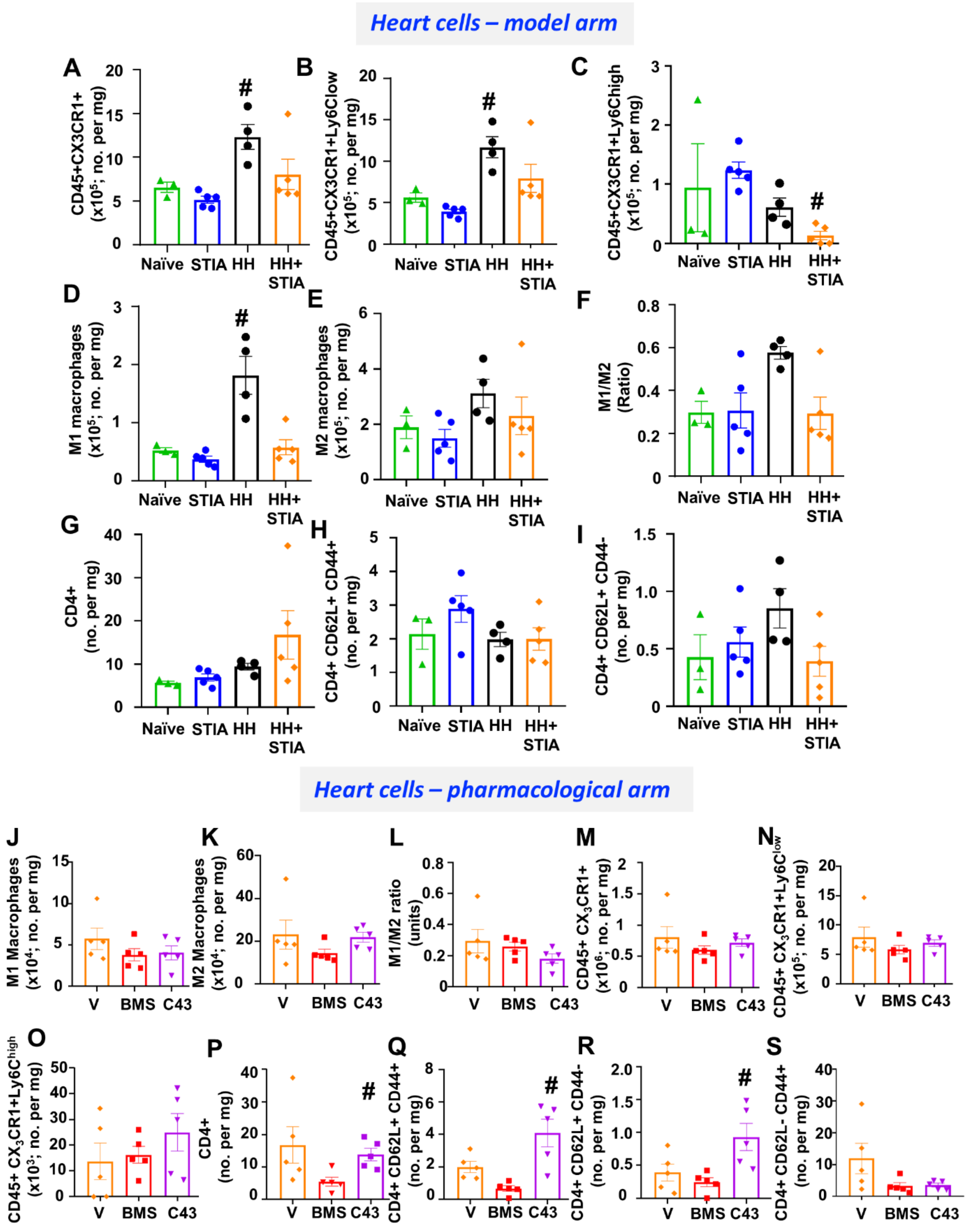

Appendix Fig. S2. Heart cell characterisation through flow cytometry analyses of heart digests. (A-I) Model arm. Data are mean±SEM of n=3 (naïve), n=4 (HH), n=5 (STIA, HH+STIA). #p<0.05 *vs.* STIA group. (J-S) Pharmacological arm: # p<0.05 *vs.* BMS group.

Lung cells – model arm

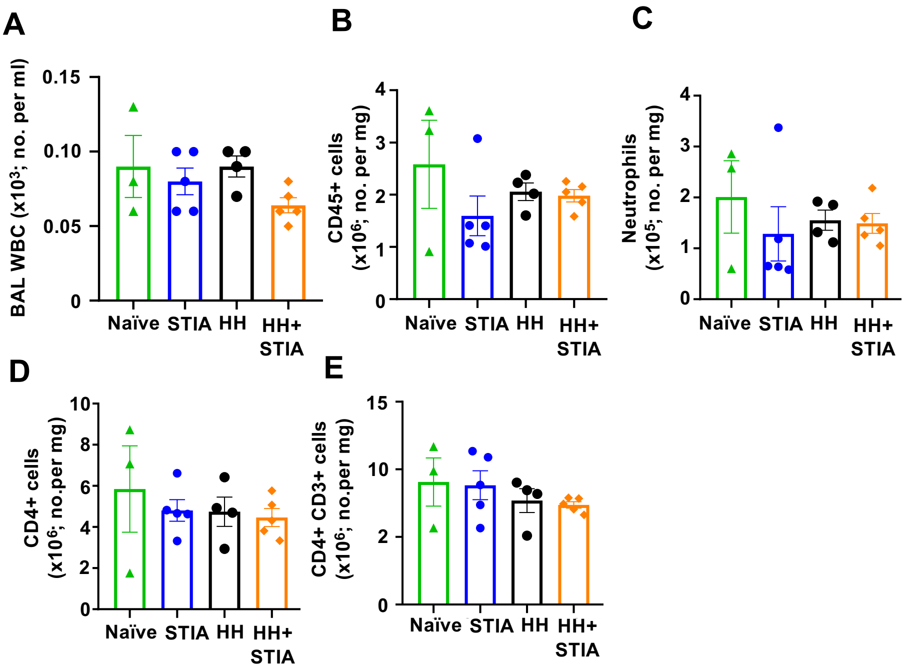

Lung cells – pharmacological arm

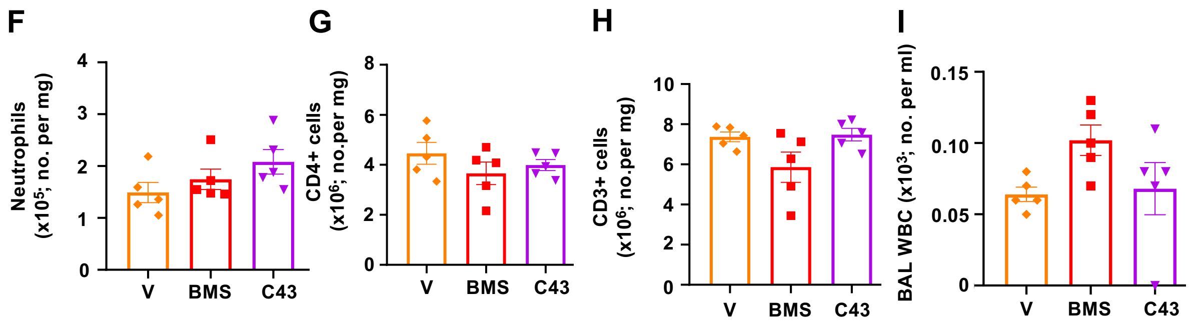

Appendix Fig. S3. Lung cell characterisation through flow cytometry analyses of lung digests. (A-E) Model arm. Data are mean±SEM of n=3 (naïve), n=4 (HH), n=5 (STIA, HH+STIA). (F-I) Pharmacological arm. Data are mean±SEM of n=5 mice per group.

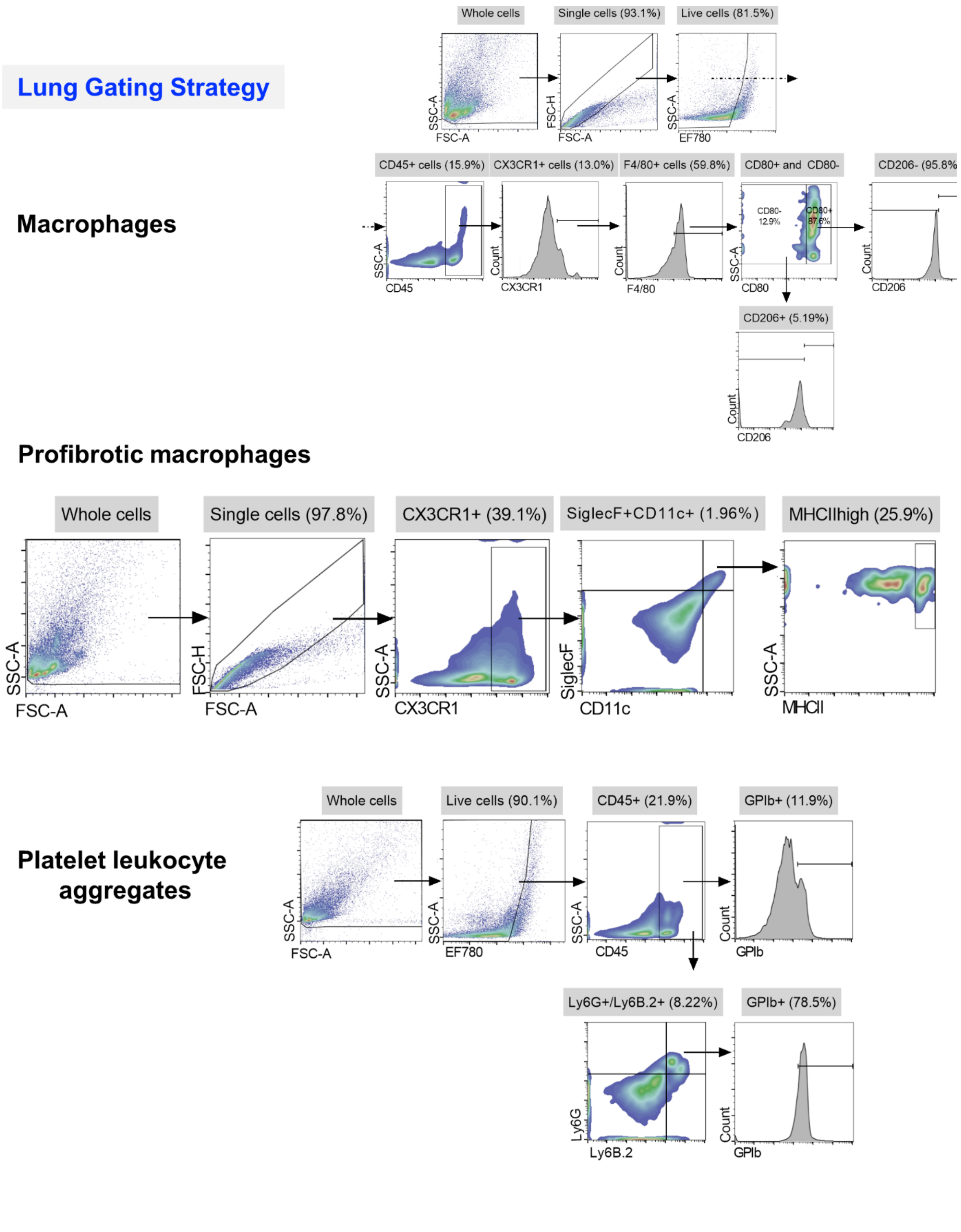

Appendix Fig. S4. Flow cytometry gating strategy for lung cells.

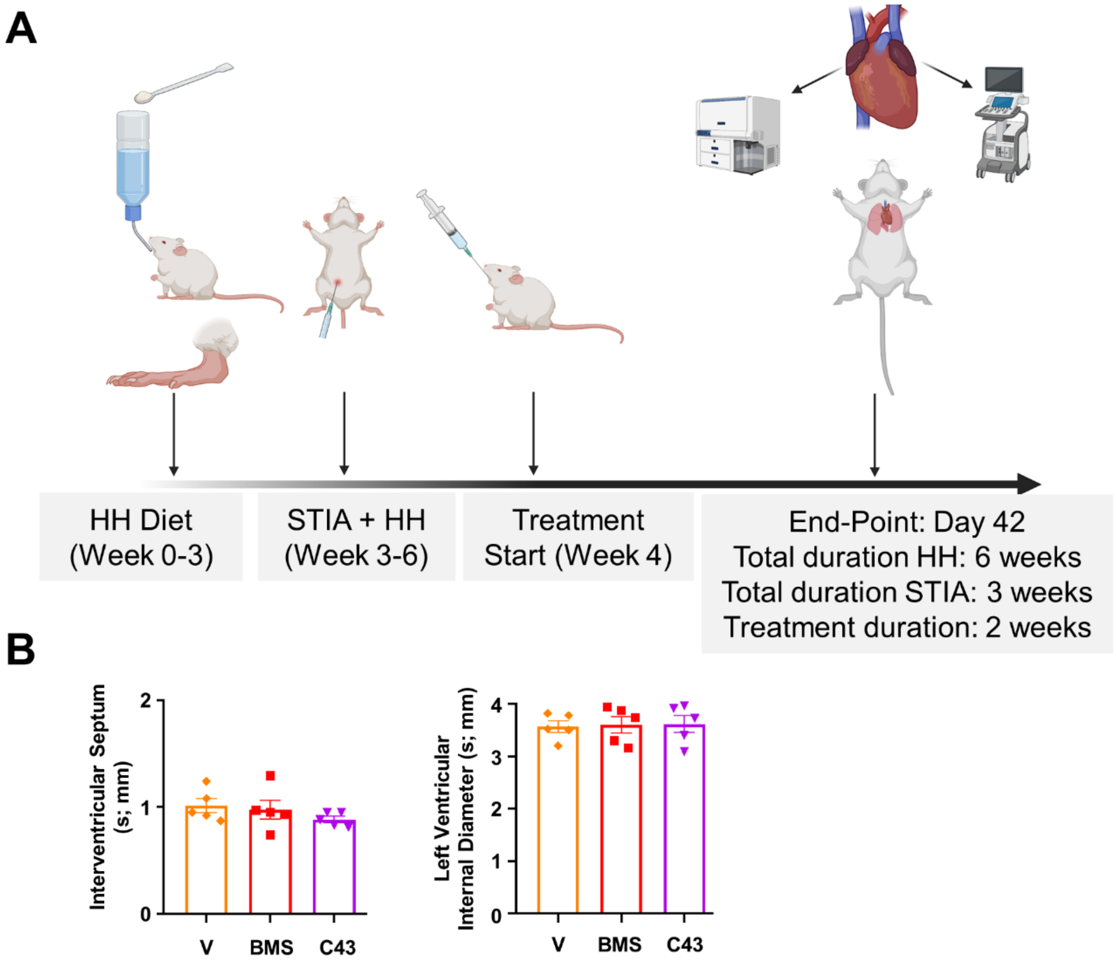

Appendix Fig. S5. (A) Schematic of the timeline for the pharmacological study. (B) Additional echocardiography parameters. Data are mean±SEM of n=5 mice per group.

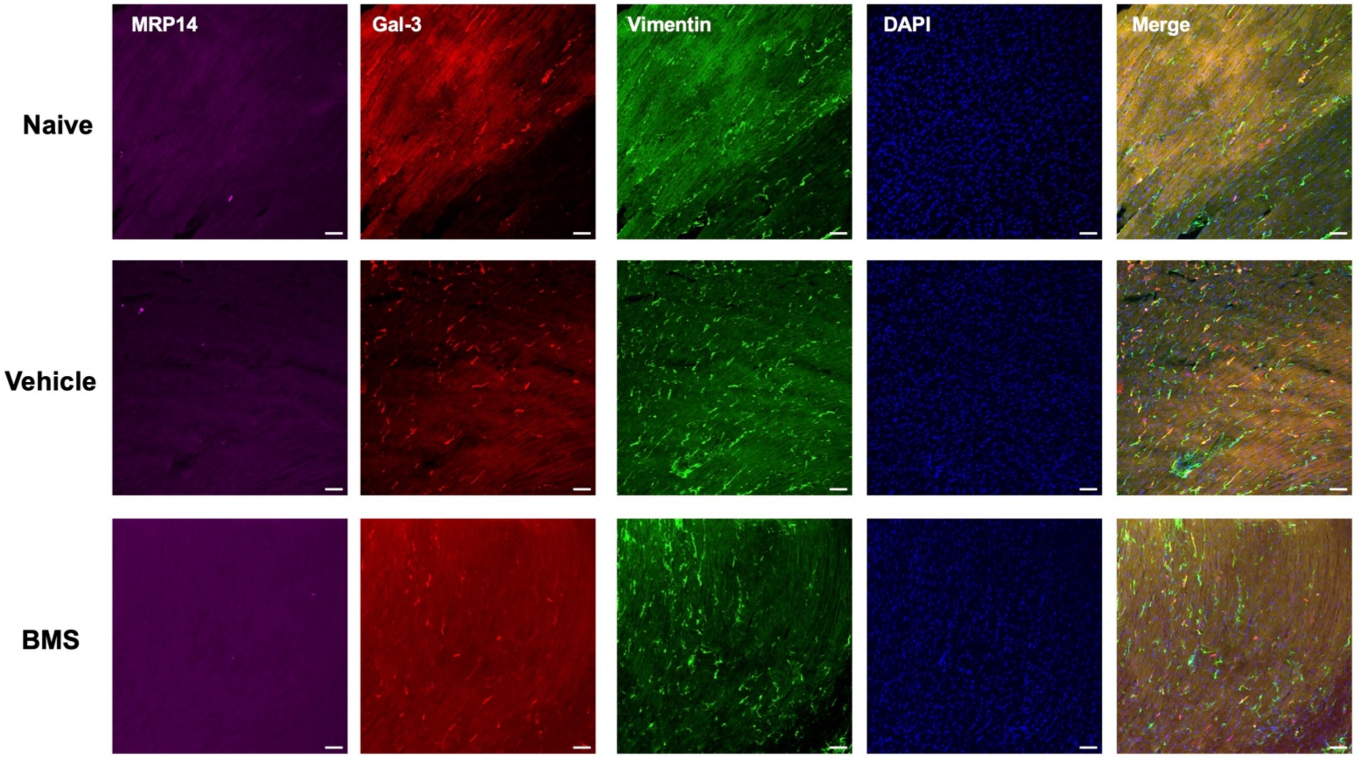

Appendix Fig. S6. Individual channels for immunofluorescence of the heart. HH+STIA was induced as in Figure 1. From week 4, HH+STIA mice were treated with either vehicle or BMS235 (3 mg/kg per os) daily and analyses were conducted at week 6 (day 42). Visualization of neutrophils, macrophages and fibroblasts in hearts using spinning disk confocal microscopy. Magenta: MRP14; red: Galectin-3; green: vimentin; blue: DAPI. Scale bar = 50 µm. Data are representative of analyses from n=4 mice per group. (N.B. the composite images are re-used from Fig 7I).

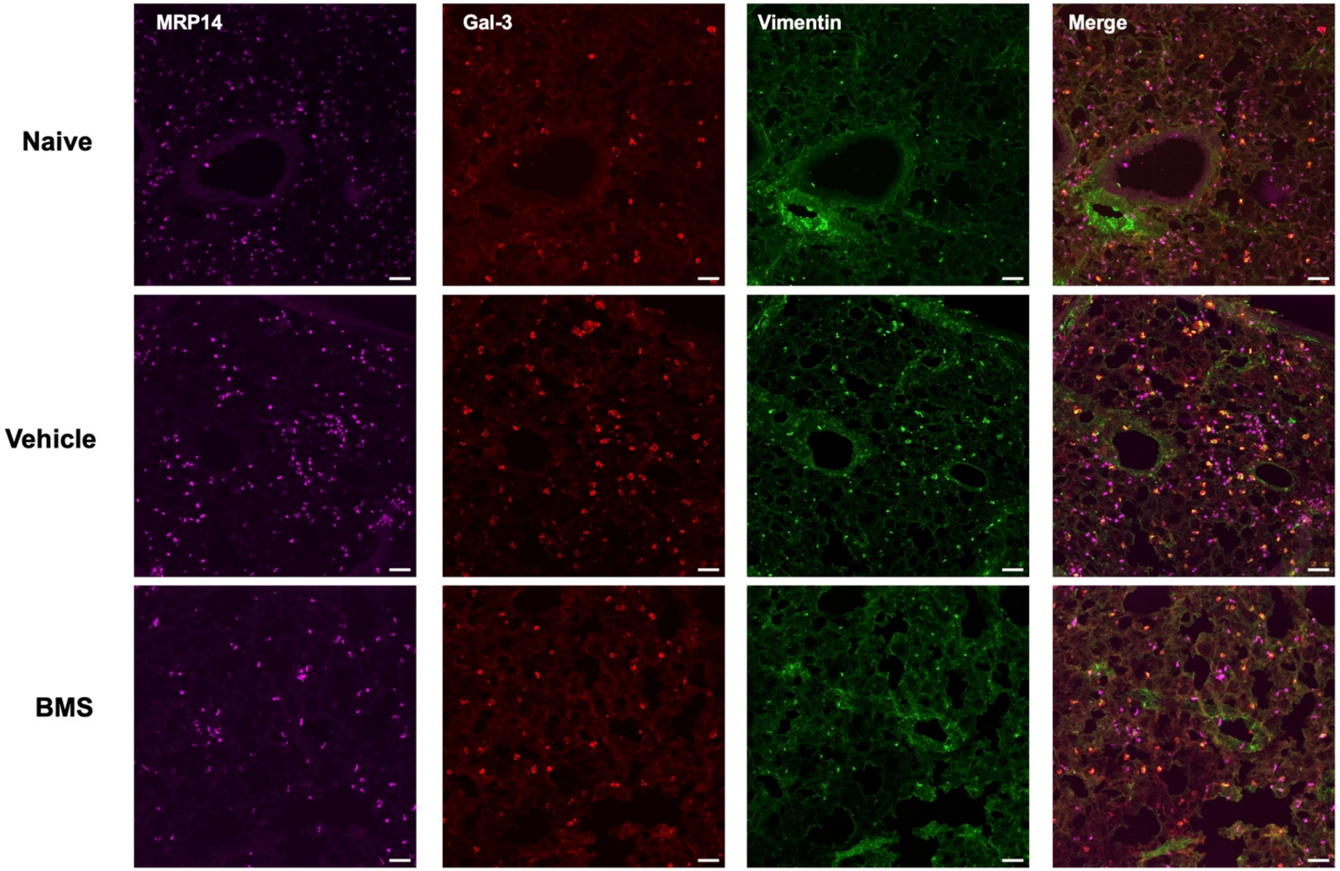

Appendix Fig. S7. Individual channels for immunofluorescence of the heart. HH+STIA was induced as in Figure 1. From week 4, HH+STIA mice were treated with either vehicle or BMS235 (3 mg/kg per os) daily and analyses were conducted at week 6 (day 42). Visualization of macrophages and neutrophils in lungs using spinning disk confocal microscopy. Magenta: MRP14; red: Galectin-3; green: vimentin. Scale bar = 50 µm. Data are representative of analyses from n=4 mice per group (N.B. the composite images are re-used from Fig 7L).

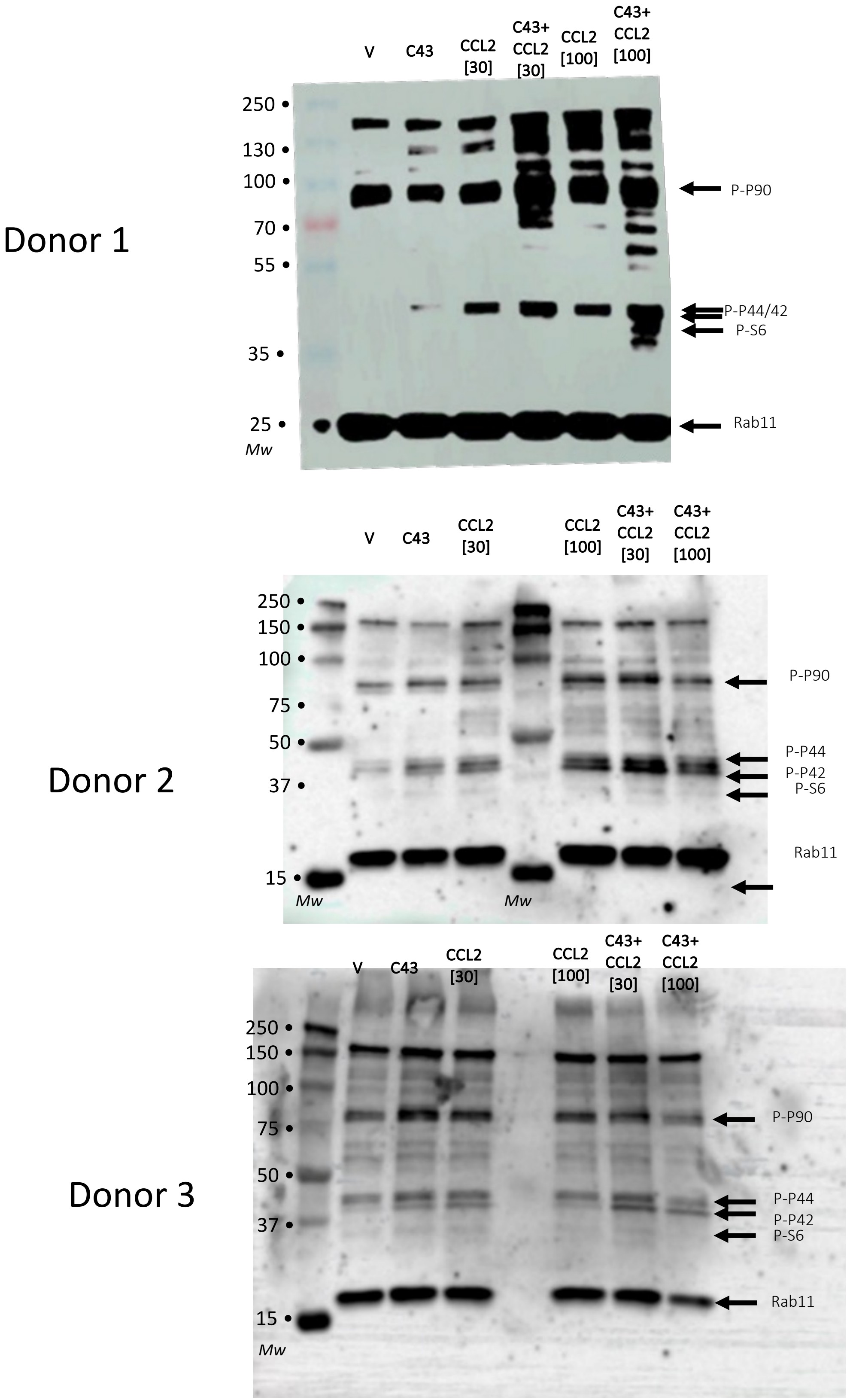

Appendix Fig. S8. PathScan™ Western blot phosphorylation screening on PBMCs treated with 30 nM C43 and the two different concentrations of CCL2. Blots from three different preparations/experiments are presented.

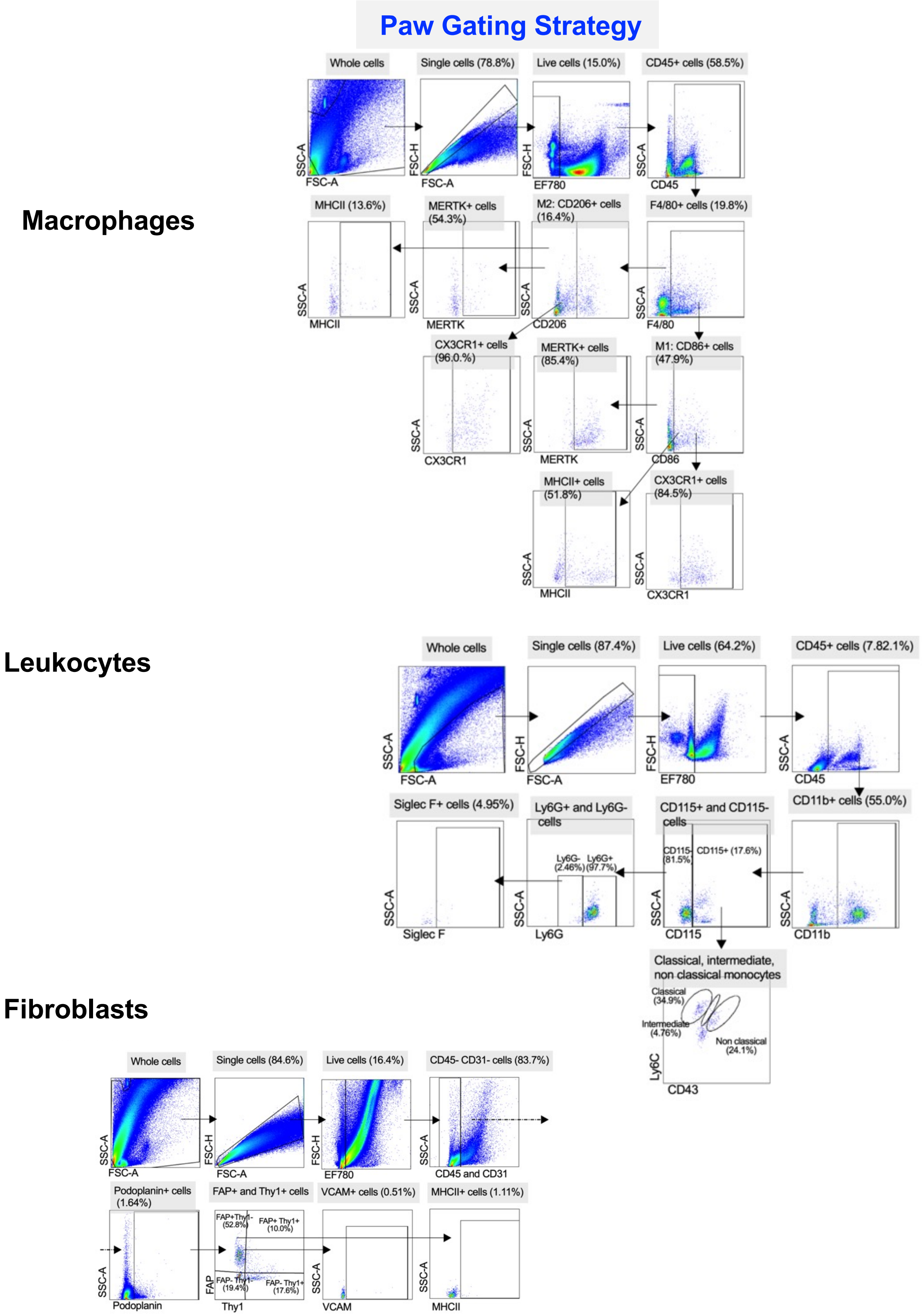

Appendix Fig. S9. Flow cytometry gating strategy for joint cells.
