# Supplementary figures and images for: Formyl-peptide receptor type 2 activation mitigates heart and lung damage in inflammatory arthritis
Source: EMBO Mol Med. 2025 Apr 3;17(5):1153–83. doi: 10.1038/s44321-025-00227-1 (PMC12081931; doi:10.1038/s44321-025-00227-1)

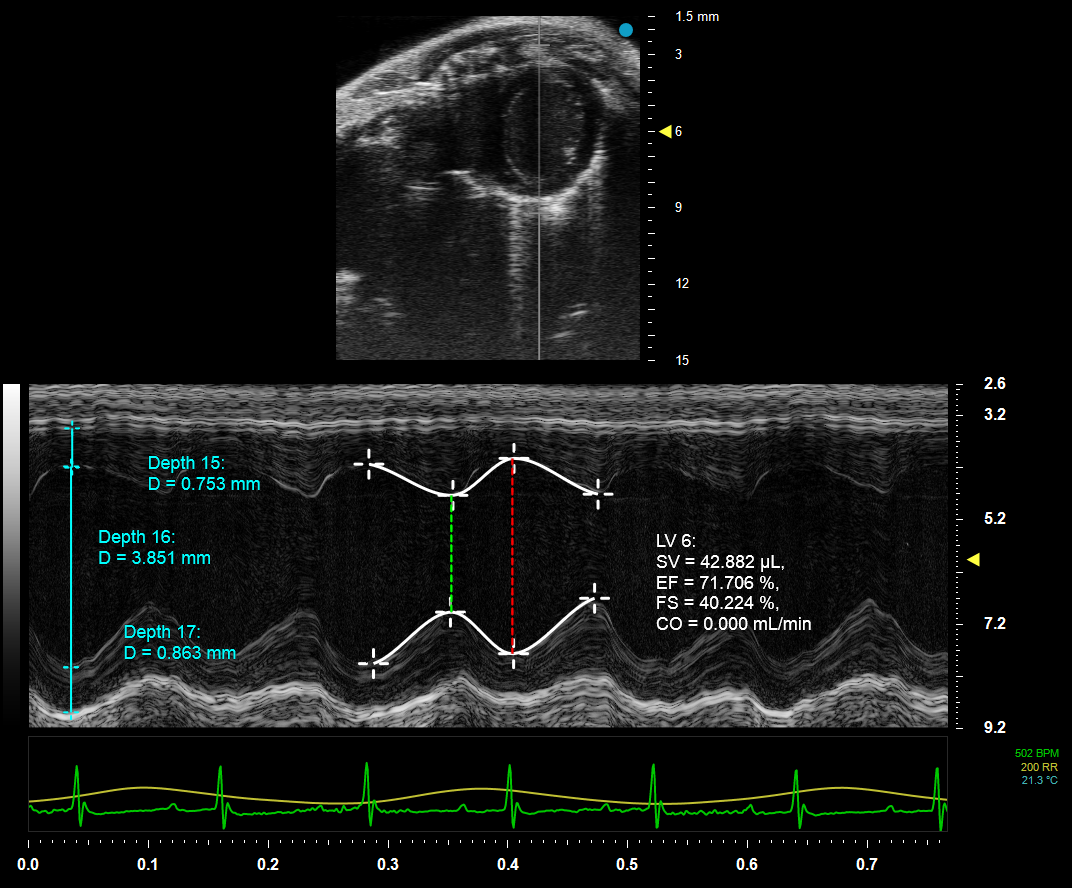

Supplement: Supplementary file 6 — Figure 2A Echo raw data part 1 [file 44321_2025_227_MOESM6_ESM.zip › Fig2 Echo pt 1/6-3 3w/20220620103154269/20220620103805394.png]

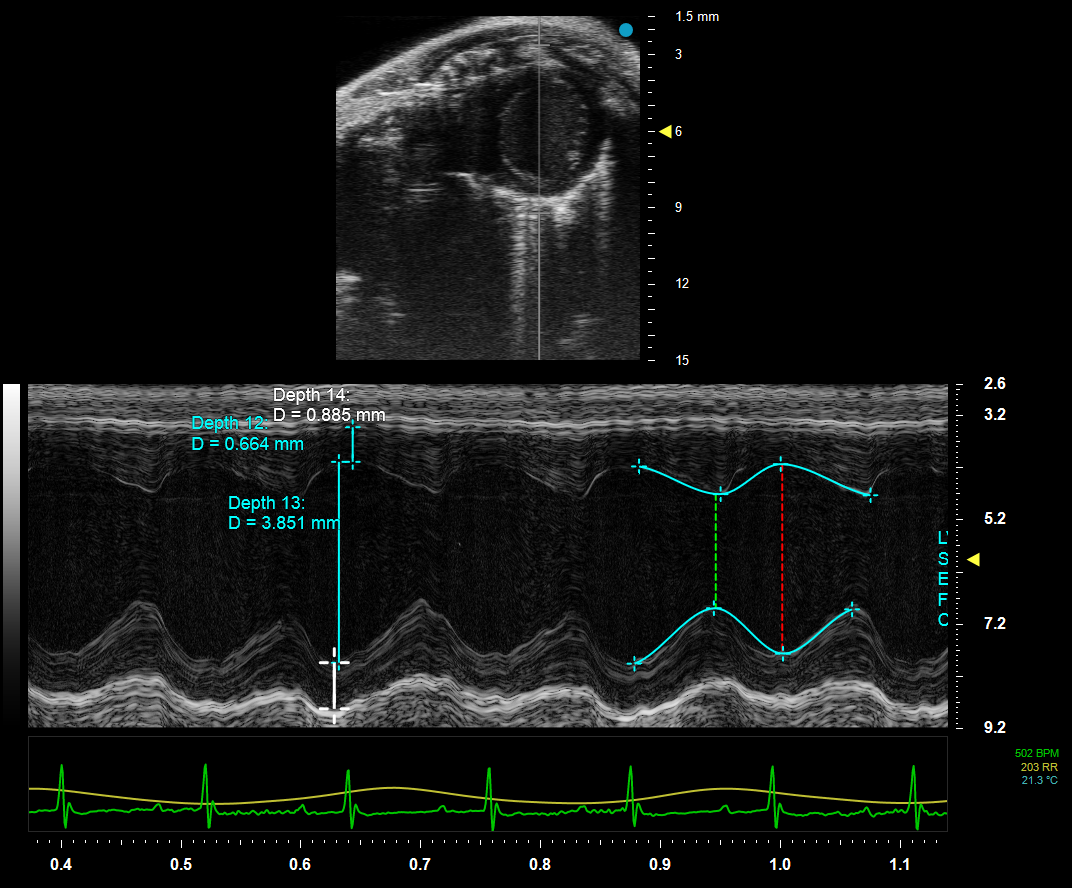

Supplement: Supplementary file 6 — Figure 2A Echo raw data part 1 [file 44321_2025_227_MOESM6_ESM.zip › Fig2 Echo pt 1/6-3 3w/20220620103154269/20220620103808144.png]

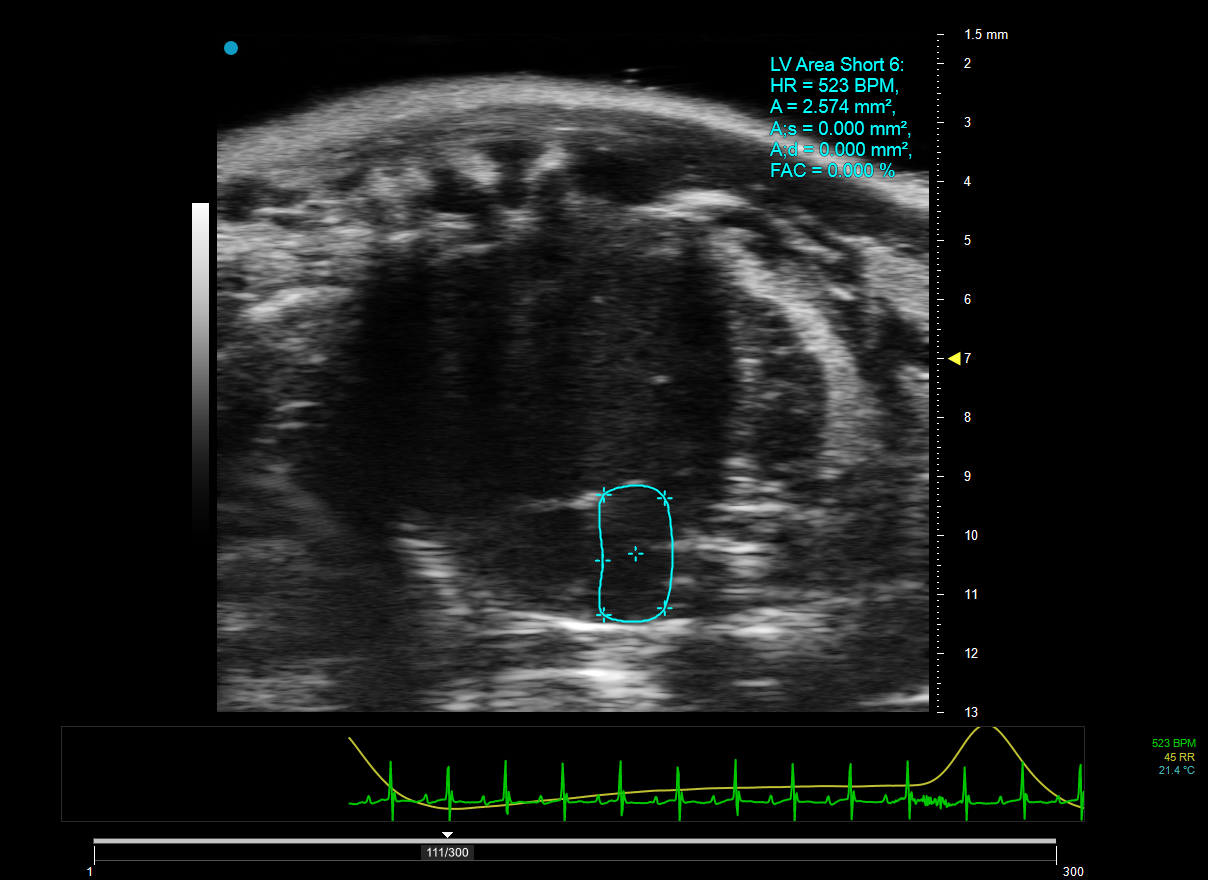

Supplement: Supplementary file 6 — Figure 2A Echo raw data part 1 [file 44321_2025_227_MOESM6_ESM.zip › Fig2 Echo pt 1/6-3 3w/20220620103154269/20220620104405486.png]

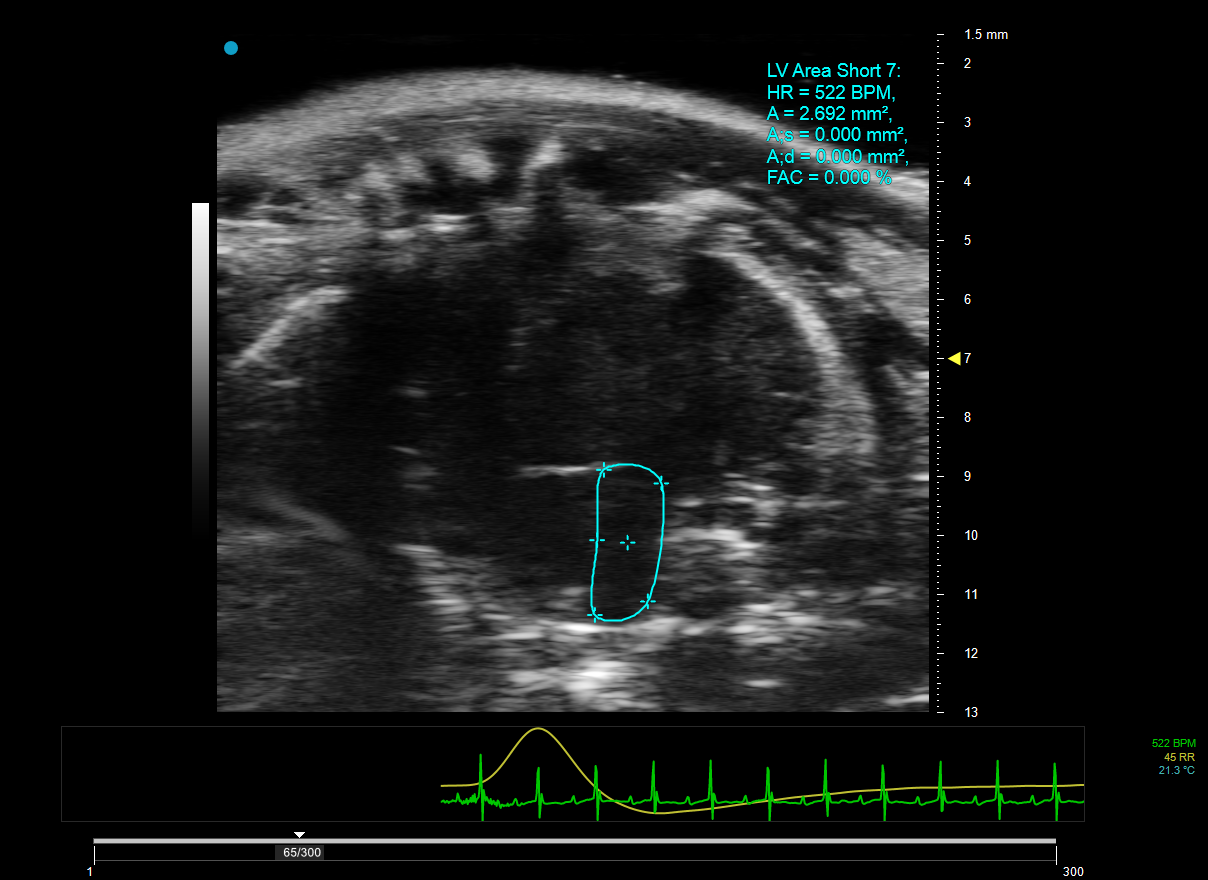

Supplement: Supplementary file 6 — Figure 2A Echo raw data part 1 [file 44321_2025_227_MOESM6_ESM.zip › Fig2 Echo pt 1/6-3 3w/20220620103154269/20220620104520662.png]

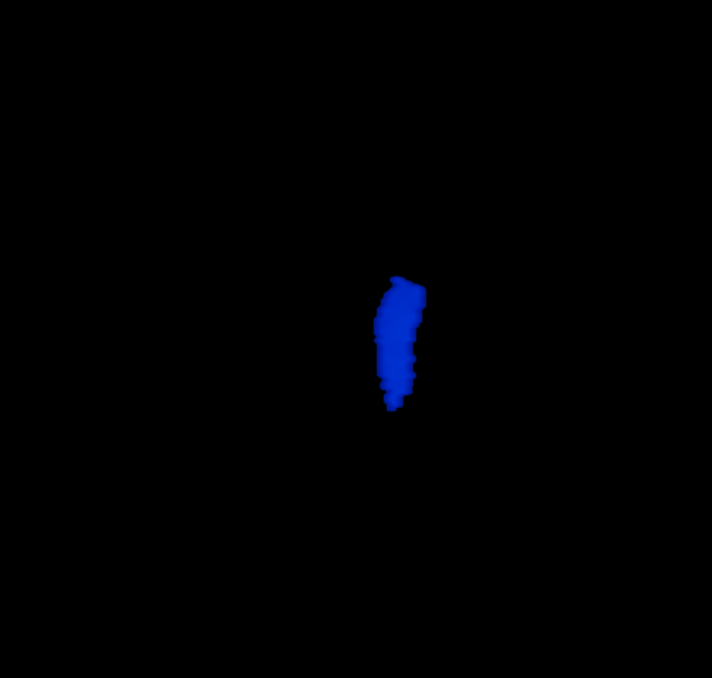

Supplement: Supplementary file 6 — Figure 2A Echo raw data part 1 [file 44321_2025_227_MOESM6_ESM.zip › Fig2 Echo pt 1/6-3 3w/20220620103154269/20220620105327359.overlay.bmp]

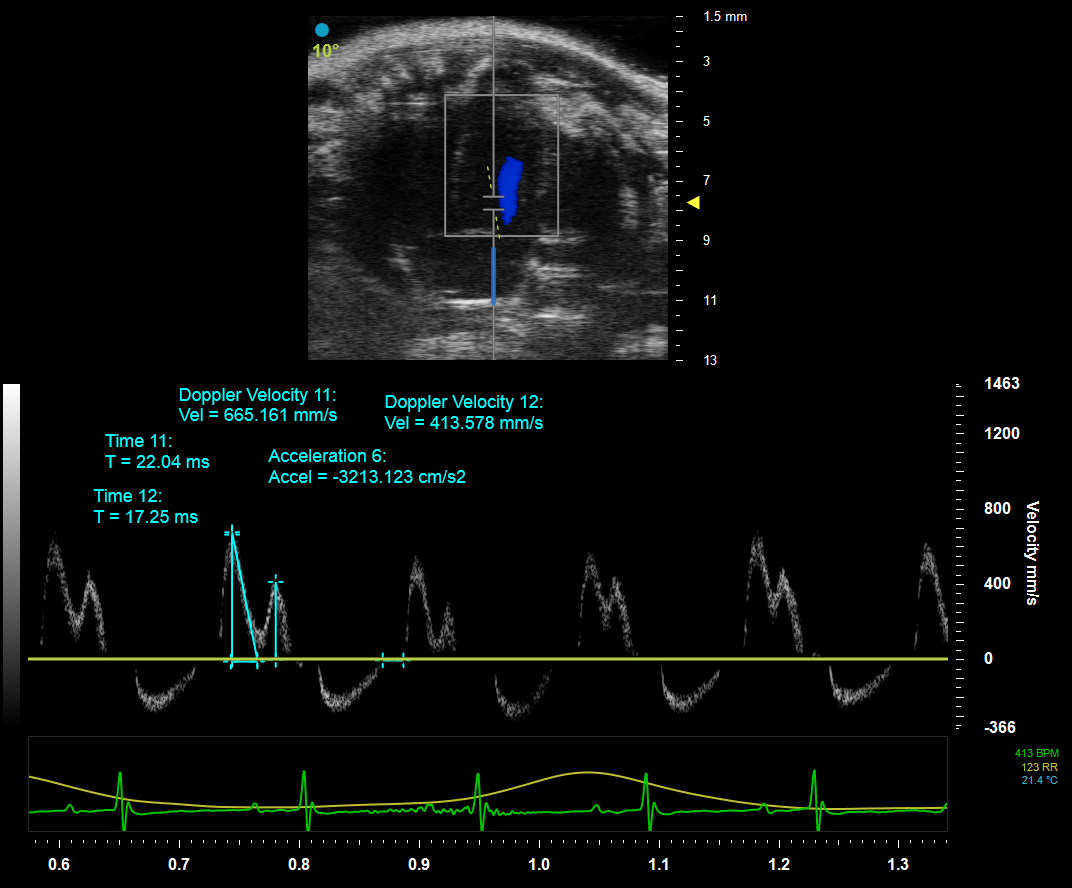

Supplement: Supplementary file 6 — Figure 2A Echo raw data part 1 [file 44321_2025_227_MOESM6_ESM.zip › Fig2 Echo pt 1/6-3 3w/20220620103154269/20220620105327359.png]

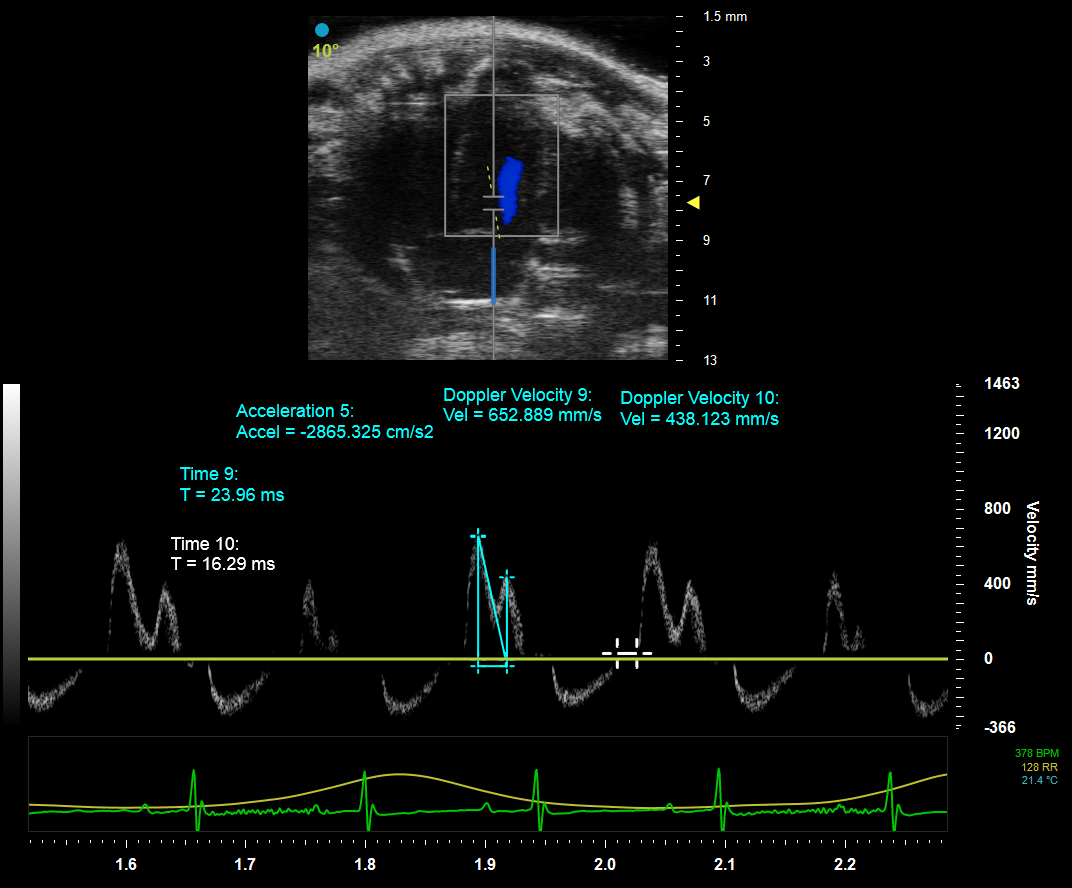

Supplement: Supplementary file 6 — Figure 2A Echo raw data part 1 [file 44321_2025_227_MOESM6_ESM.zip › Fig2 Echo pt 1/6-3 3w/20220620103154269/20220620105330620.png]

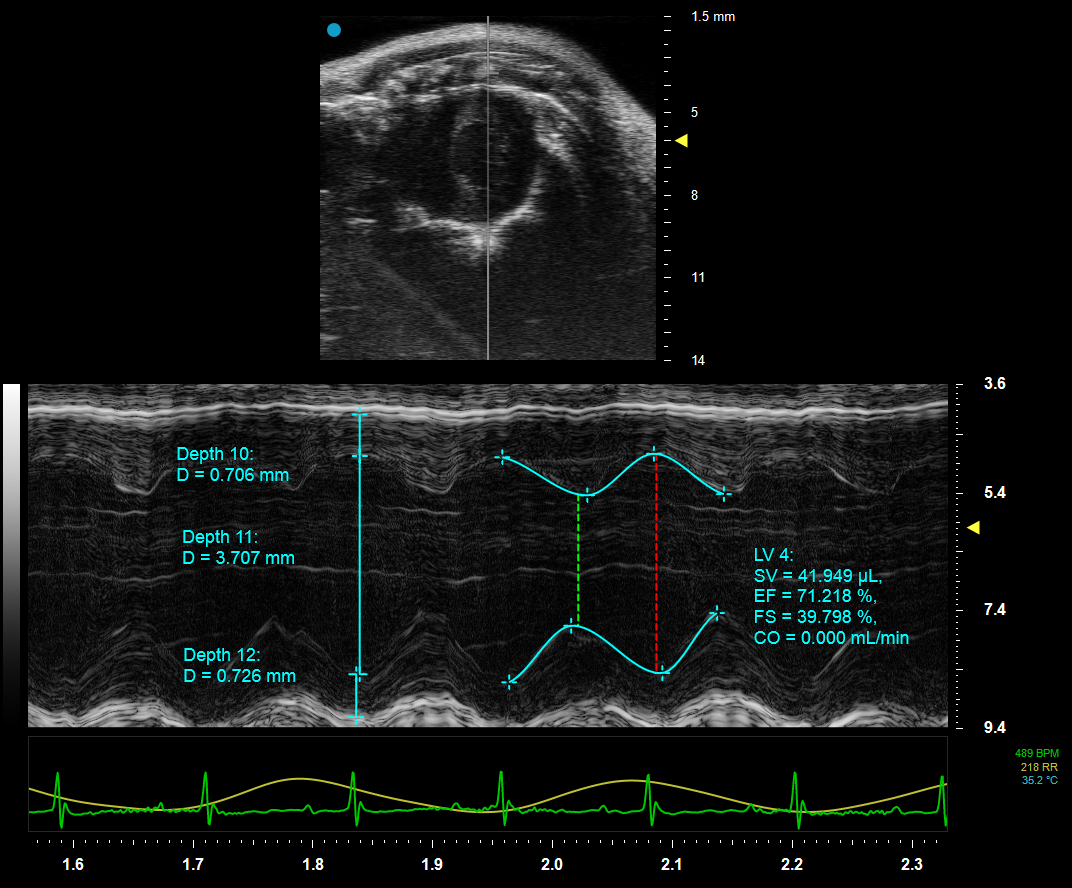

Supplement: Supplementary file 6 — Figure 2A Echo raw data part 1 [file 44321_2025_227_MOESM6_ESM.zip › Fig2 Echo pt 1/6-3 4w base/20220628102905541/20220628103131649.png]

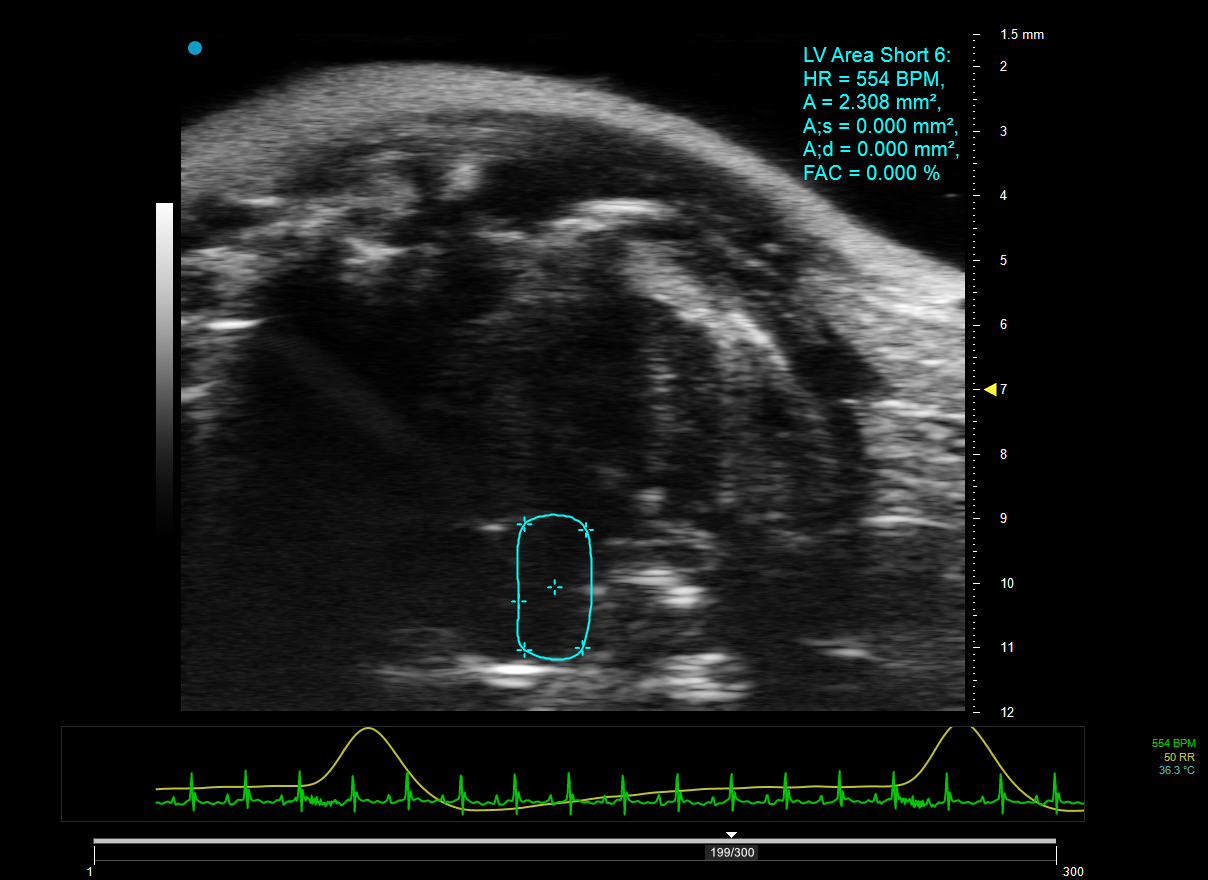

Supplement: Supplementary file 6 — Figure 2A Echo raw data part 1 [file 44321_2025_227_MOESM6_ESM.zip › Fig2 Echo pt 1/6-3 4w base/20220628102905541/20220628103807771.png]

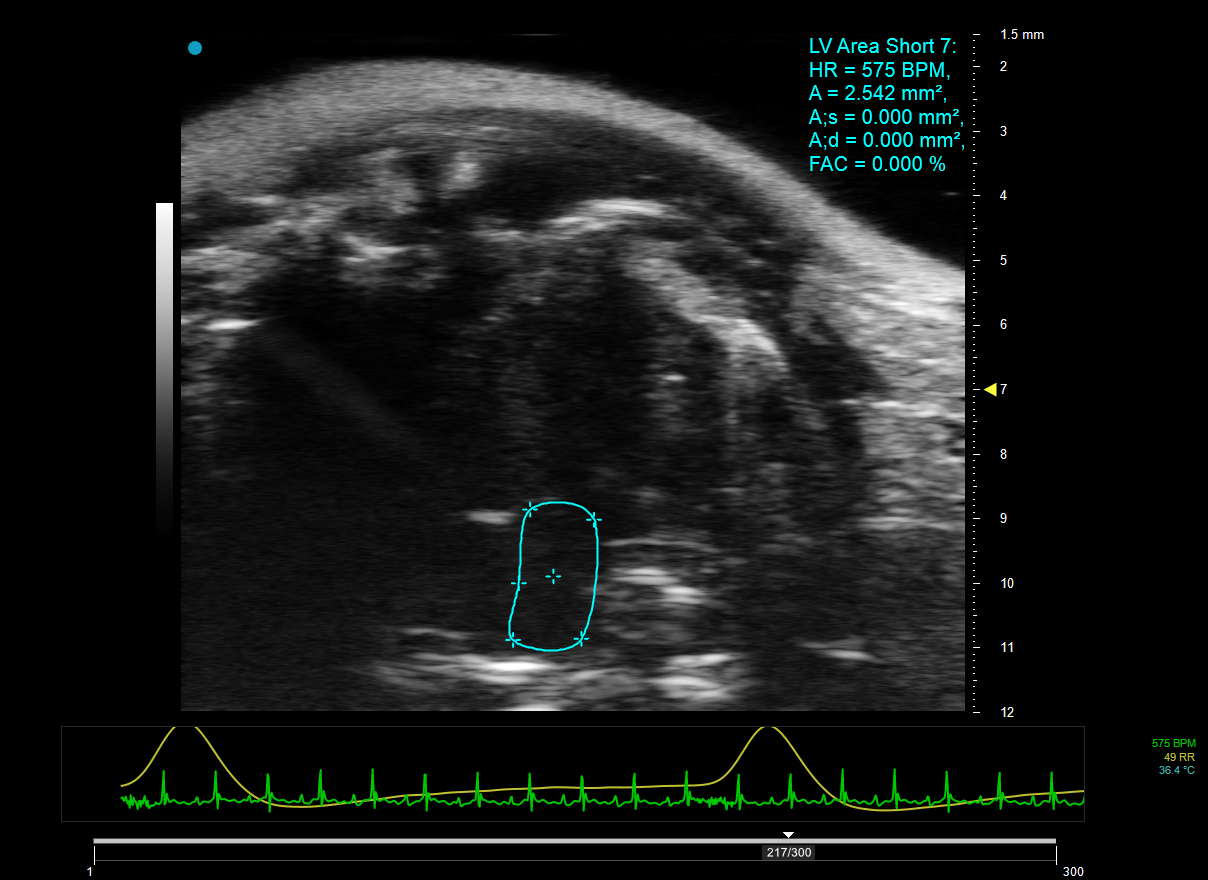

Supplement: Supplementary file 6 — Figure 2A Echo raw data part 1 [file 44321_2025_227_MOESM6_ESM.zip › Fig2 Echo pt 1/6-3 4w base/20220628102905541/20220628104026952.png]

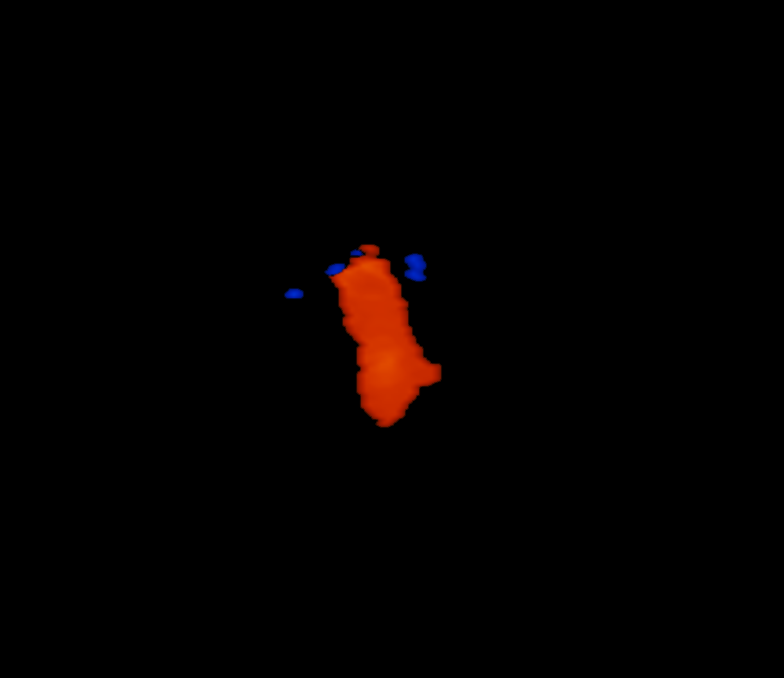

Supplement: Supplementary file 6 — Figure 2A Echo raw data part 1 [file 44321_2025_227_MOESM6_ESM.zip › Fig2 Echo pt 1/6-3 4w base/20220628102905541/20220628104440252.overlay.bmp]

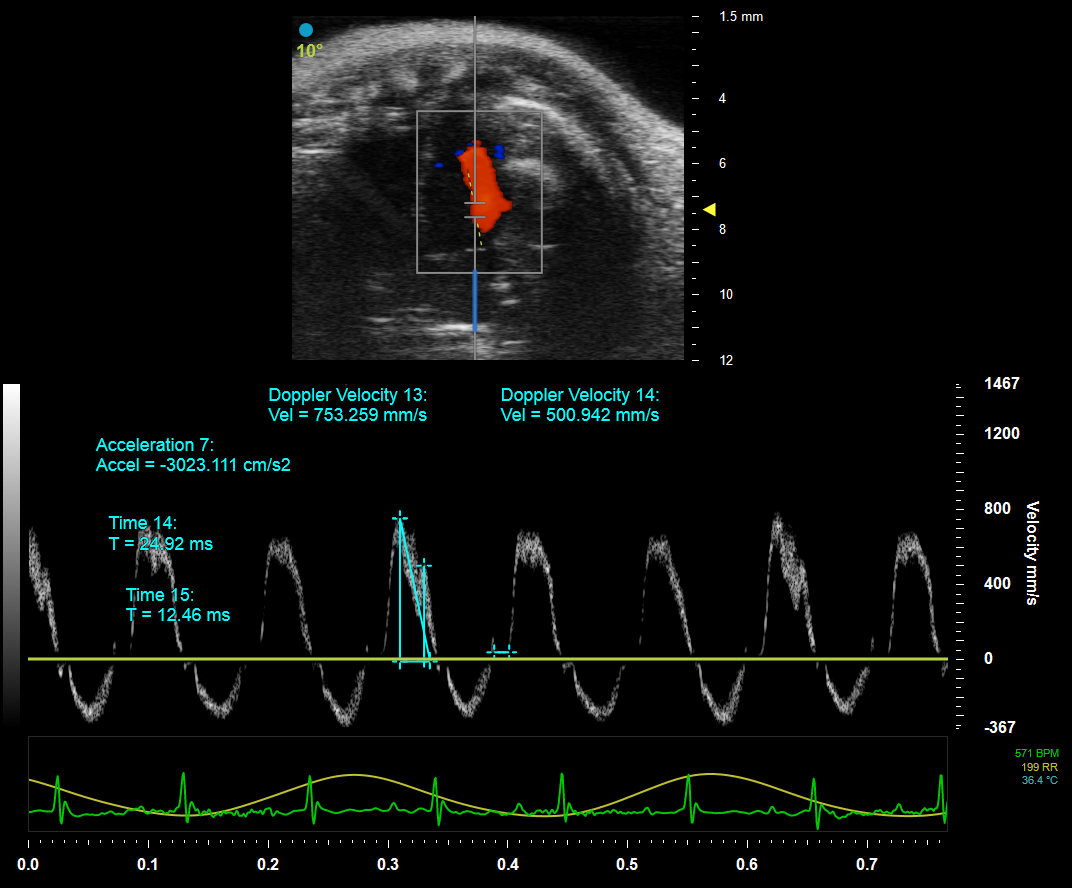

Supplement: Supplementary file 6 — Figure 2A Echo raw data part 1 [file 44321_2025_227_MOESM6_ESM.zip › Fig2 Echo pt 1/6-3 4w base/20220628102905541/20220628104440252.png]

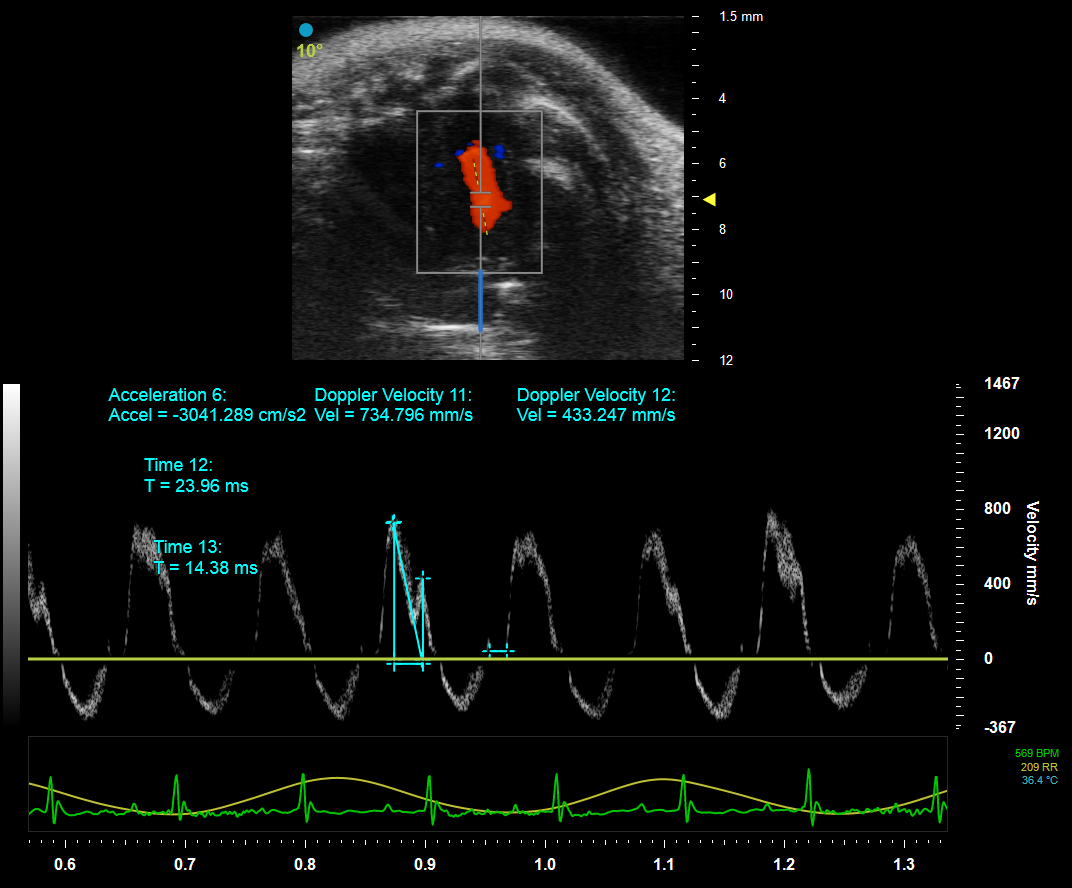

Supplement: Supplementary file 6 — Figure 2A Echo raw data part 1 [file 44321_2025_227_MOESM6_ESM.zip › Fig2 Echo pt 1/6-3 4w base/20220628102905541/20220628104454582.png]

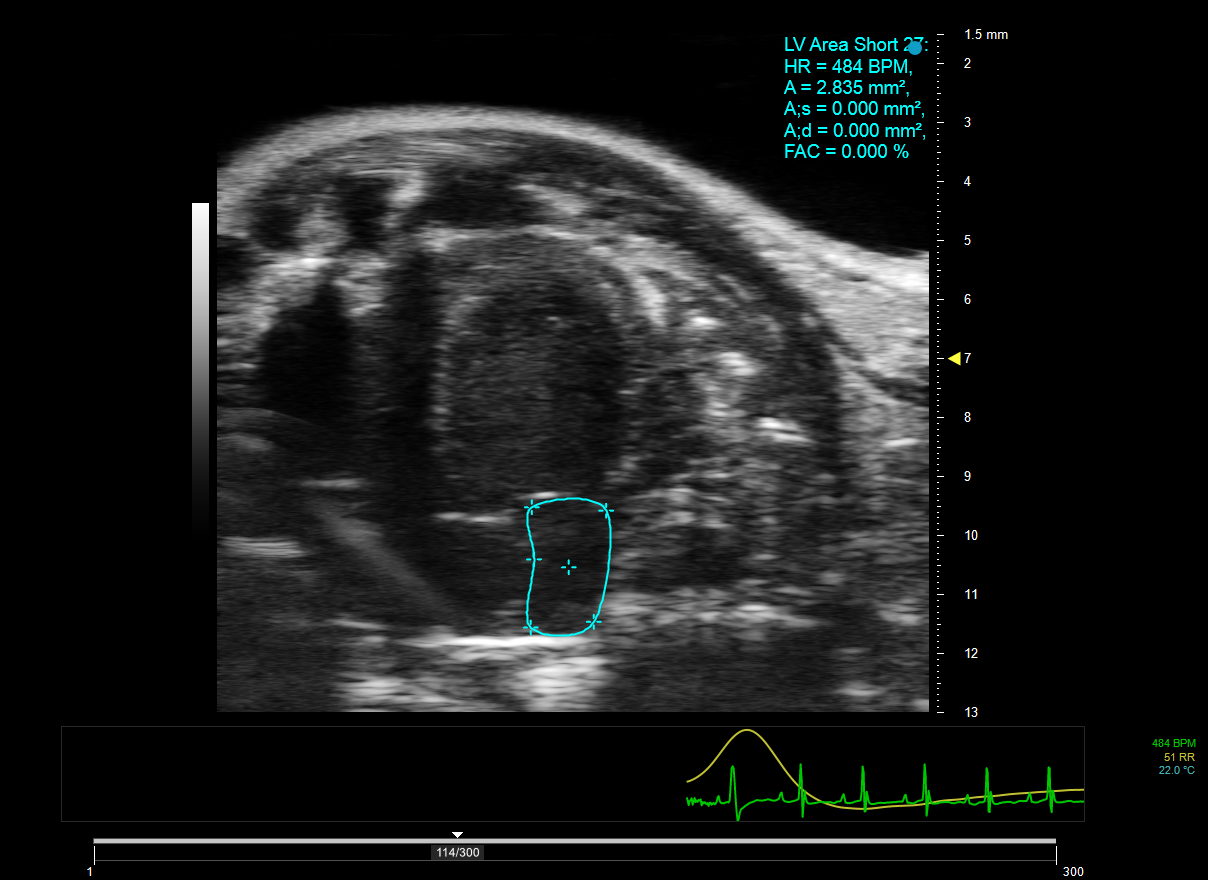

Supplement: Supplementary file 7 — Figure 2A Echo raw data part 2 [file 44321_2025_227_MOESM7_ESM.zip › Fig2 Echo pt 2/2-2 3w/20220620152946782/20220620152957563.png]

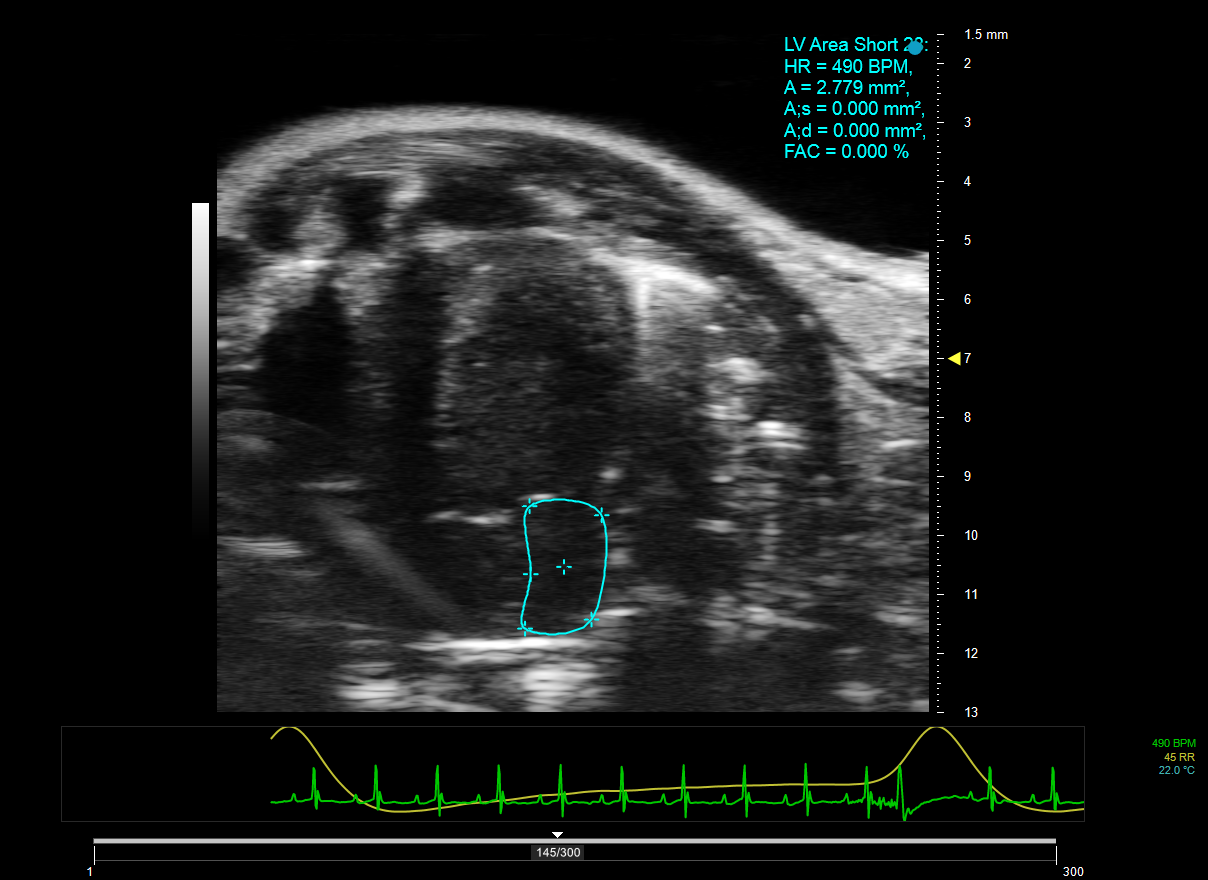

Supplement: Supplementary file 7 — Figure 2A Echo raw data part 2 [file 44321_2025_227_MOESM7_ESM.zip › Fig2 Echo pt 2/2-2 3w/20220620152946782/20220620153159797.png]

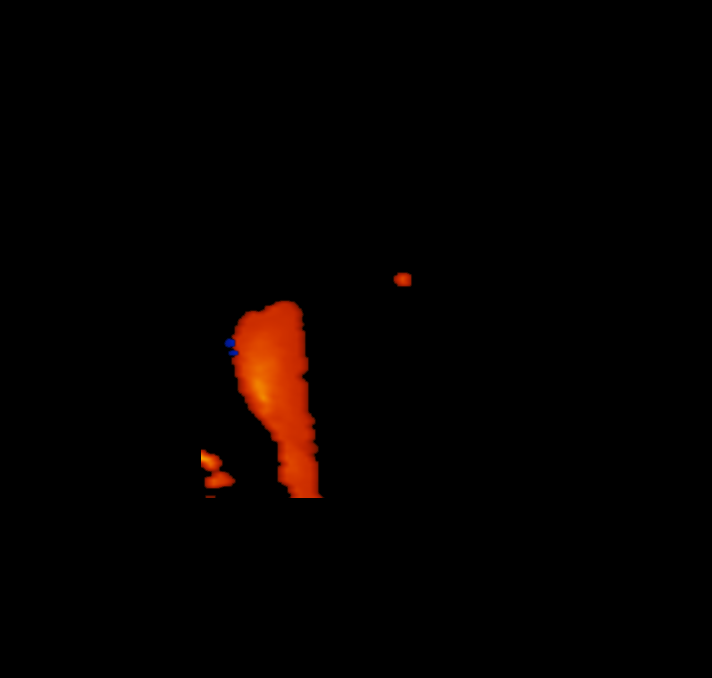

Supplement: Supplementary file 7 — Figure 2A Echo raw data part 2 [file 44321_2025_227_MOESM7_ESM.zip › Fig2 Echo pt 2/2-2 3w/20220620152946782/20220620153347512.overlay.bmp]

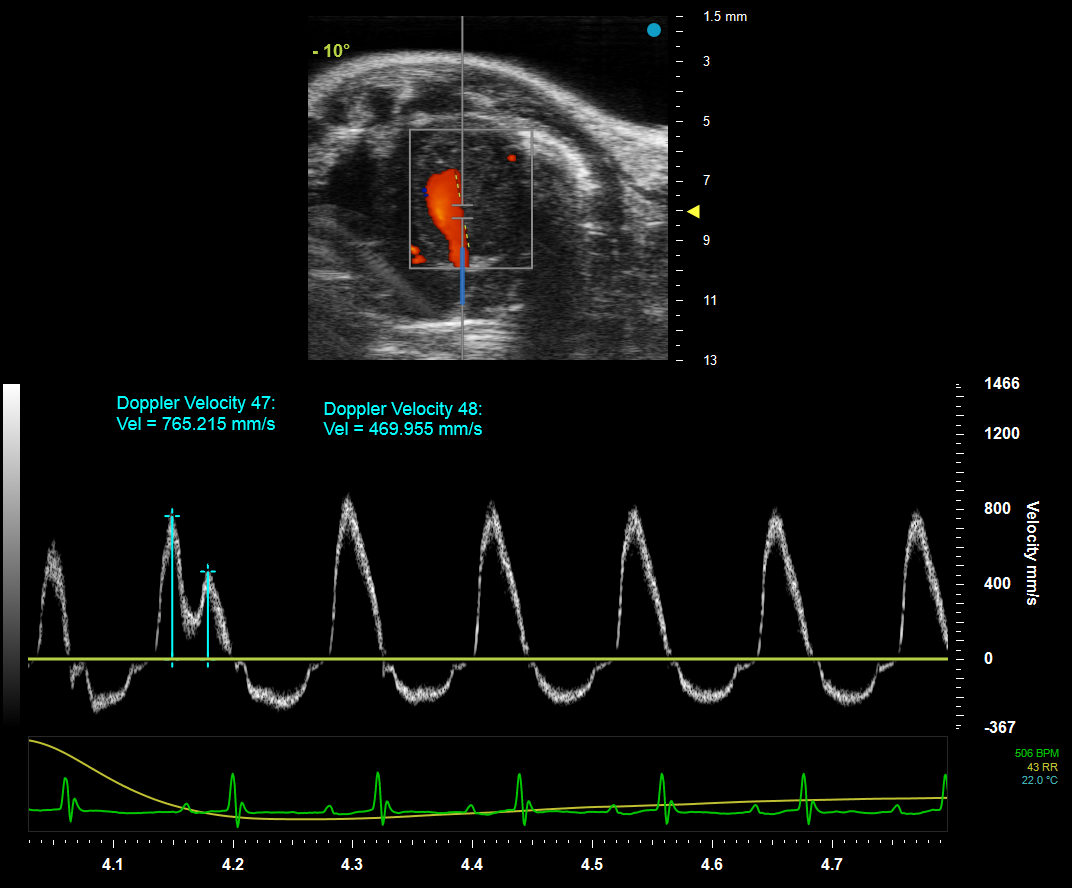

Supplement: Supplementary file 7 — Figure 2A Echo raw data part 2 [file 44321_2025_227_MOESM7_ESM.zip › Fig2 Echo pt 2/2-2 3w/20220620152946782/20220620153347512.png]

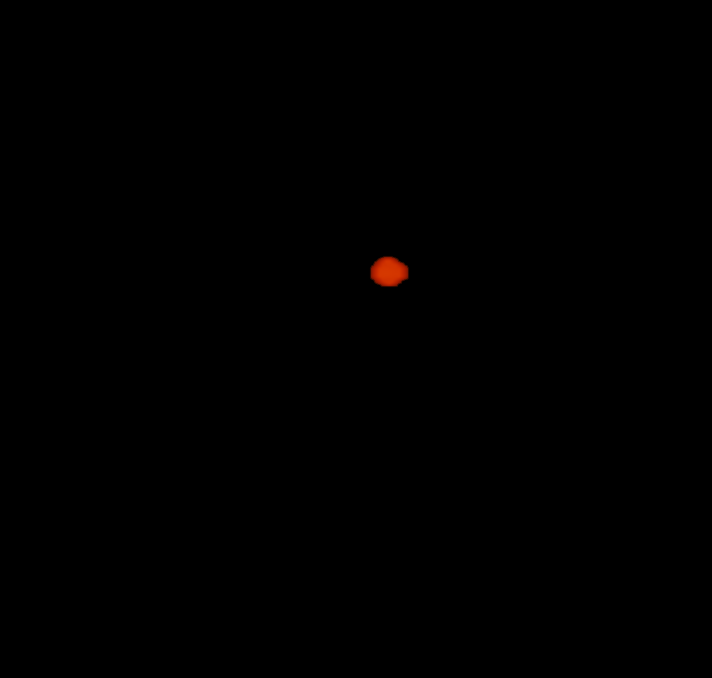

Supplement: Supplementary file 7 — Figure 2A Echo raw data part 2 [file 44321_2025_227_MOESM7_ESM.zip › Fig2 Echo pt 2/2-2 3w/20220620152946782/20220620153525270.overlay.bmp]

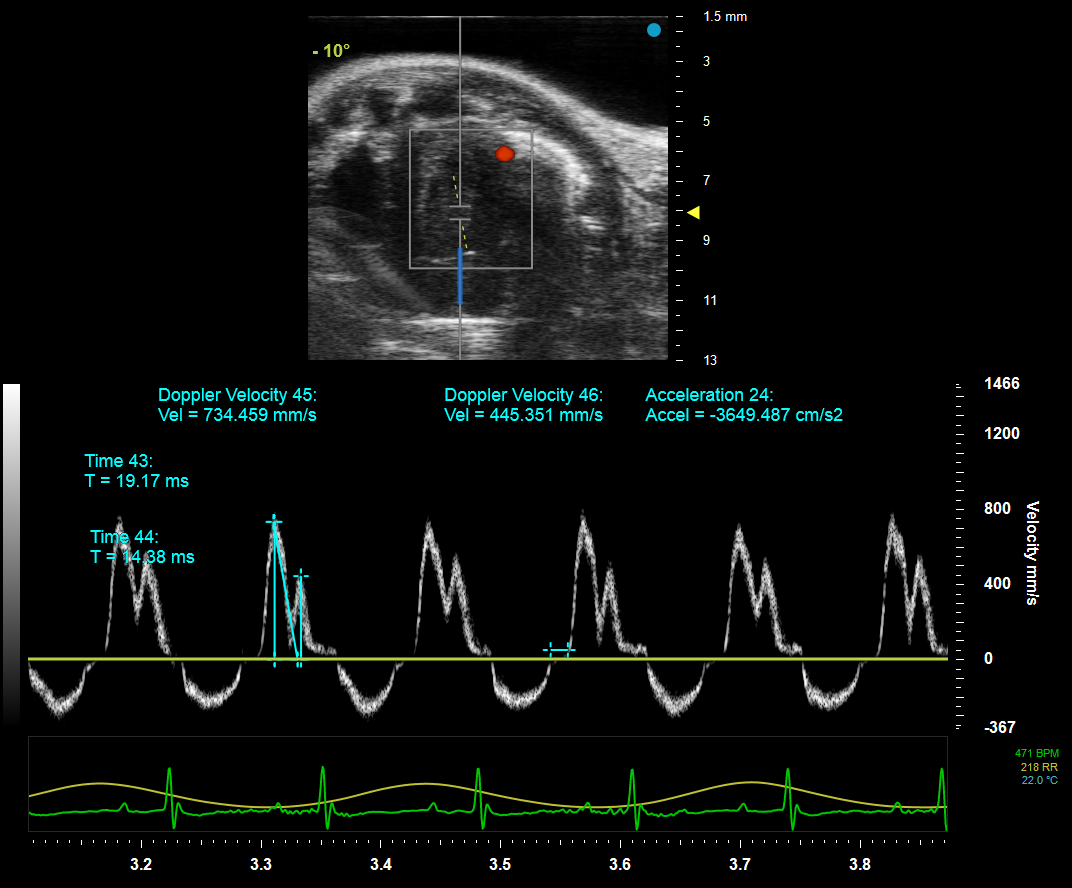

Supplement: Supplementary file 7 — Figure 2A Echo raw data part 2 [file 44321_2025_227_MOESM7_ESM.zip › Fig2 Echo pt 2/2-2 3w/20220620152946782/20220620153525270.png]

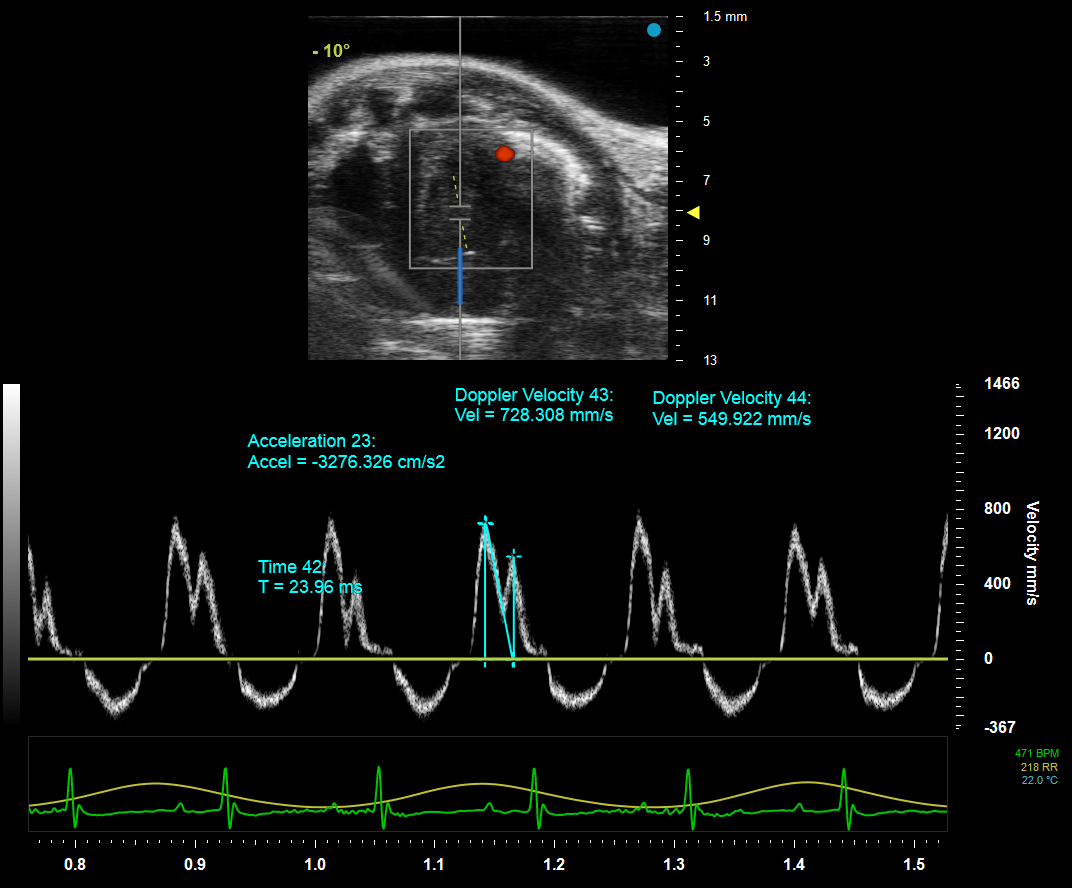

Supplement: Supplementary file 7 — Figure 2A Echo raw data part 2 [file 44321_2025_227_MOESM7_ESM.zip › Fig2 Echo pt 2/2-2 3w/20220620152946782/20220620153527514.png]

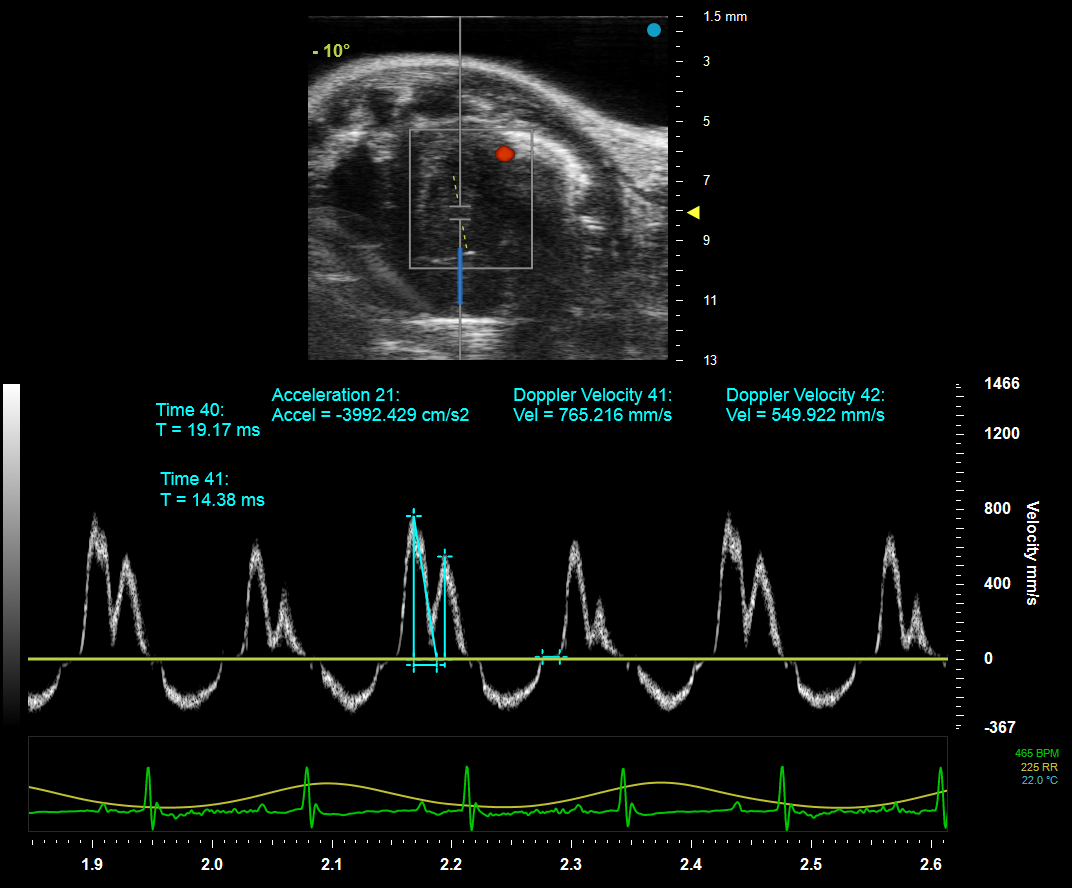

Supplement: Supplementary file 7 — Figure 2A Echo raw data part 2 [file 44321_2025_227_MOESM7_ESM.zip › Fig2 Echo pt 2/2-2 3w/20220620152946782/20220620153531388.png]

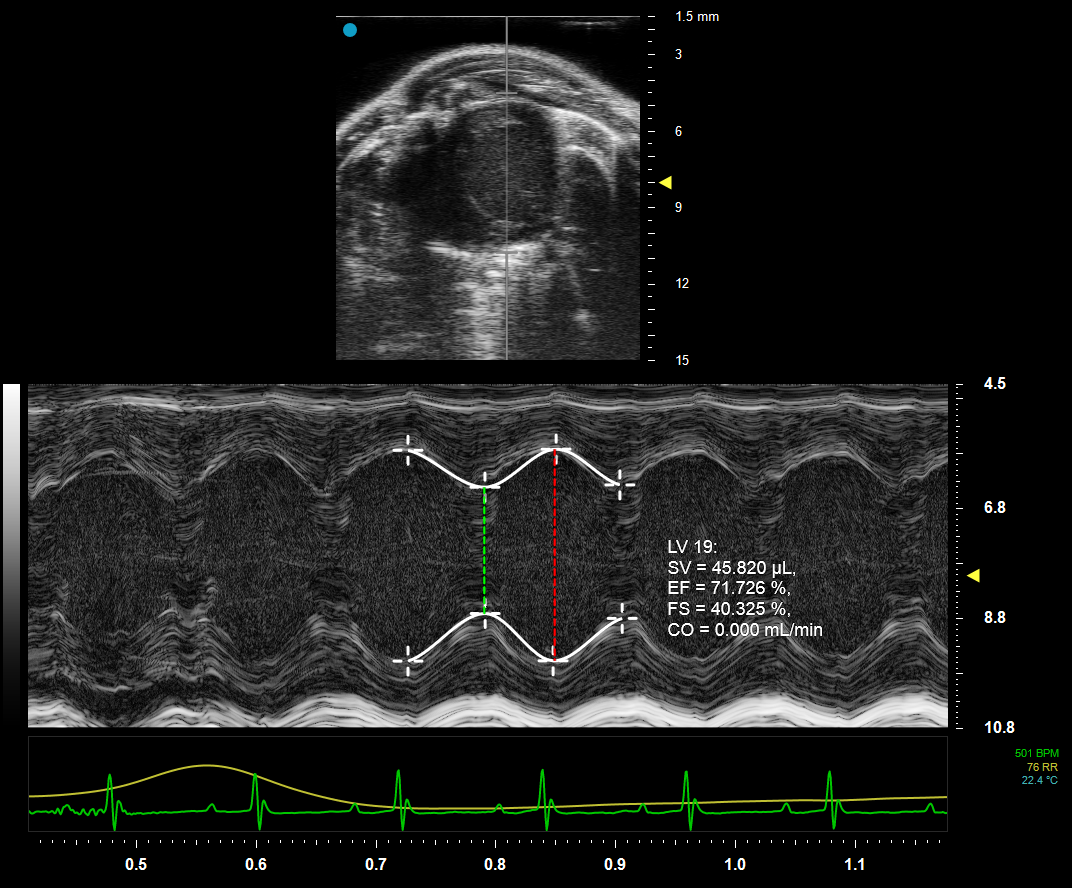

Supplement: Supplementary file 7 — Figure 2A Echo raw data part 2 [file 44321_2025_227_MOESM7_ESM.zip › Fig2 Echo pt 2/2-2 3w/20220620152946782/20220620154507262.png]

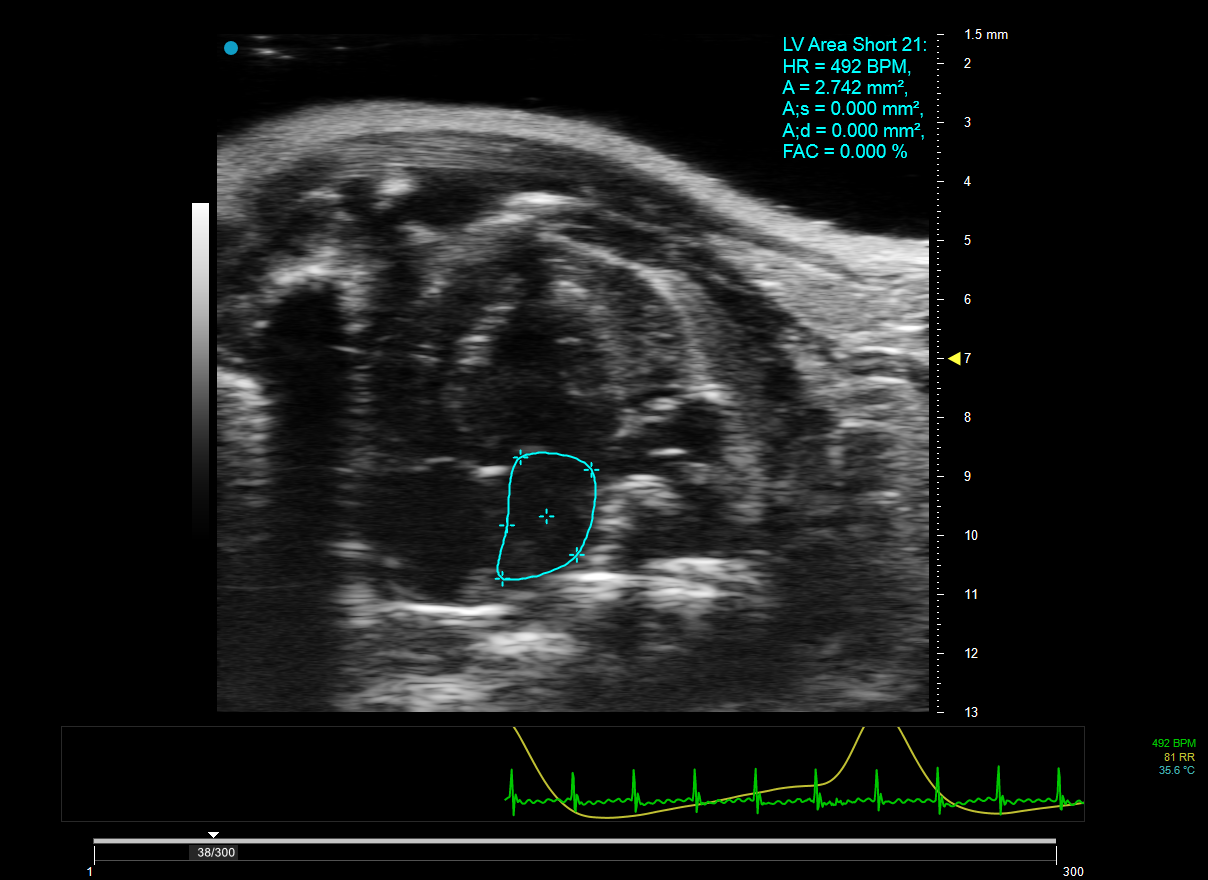

Supplement: Supplementary file 7 — Figure 2A Echo raw data part 2 [file 44321_2025_227_MOESM7_ESM.zip › Fig2 Echo pt 2/2-2 4w base/20220629091722400/20220629091758471.png]

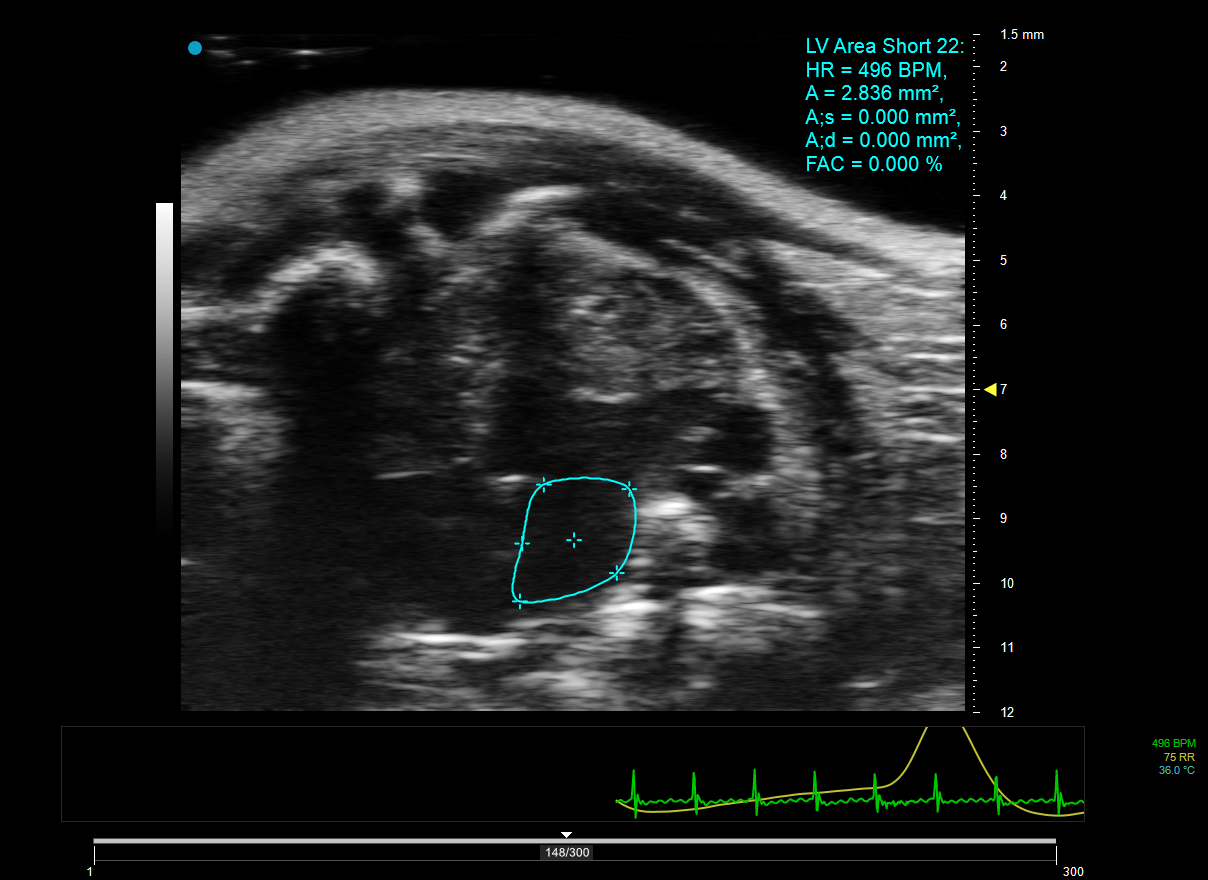

Supplement: Supplementary file 7 — Figure 2A Echo raw data part 2 [file 44321_2025_227_MOESM7_ESM.zip › Fig2 Echo pt 2/2-2 4w base/20220629091722400/20220629091950129.png]

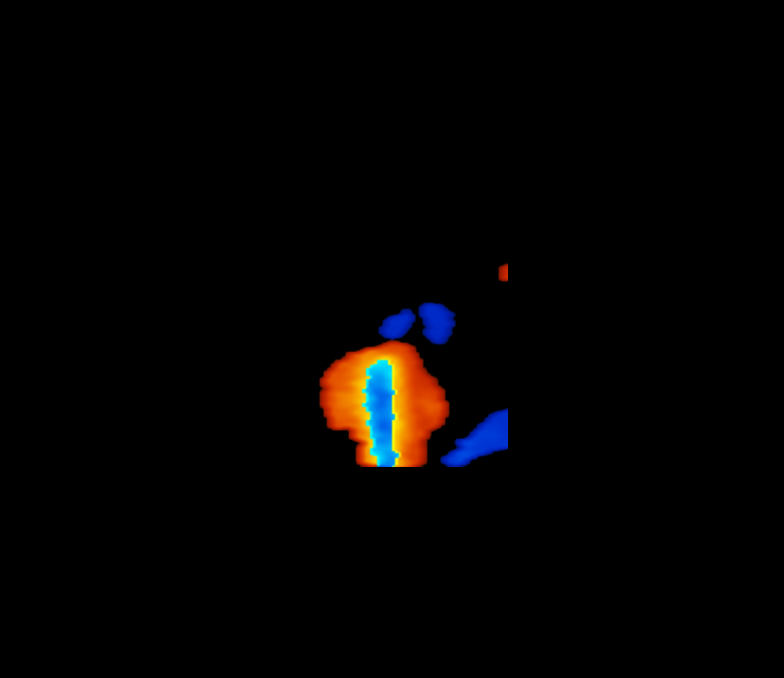

Supplement: Supplementary file 7 — Figure 2A Echo raw data part 2 [file 44321_2025_227_MOESM7_ESM.zip › Fig2 Echo pt 2/2-2 4w base/20220629091722400/20220629092251866.overlay.bmp]

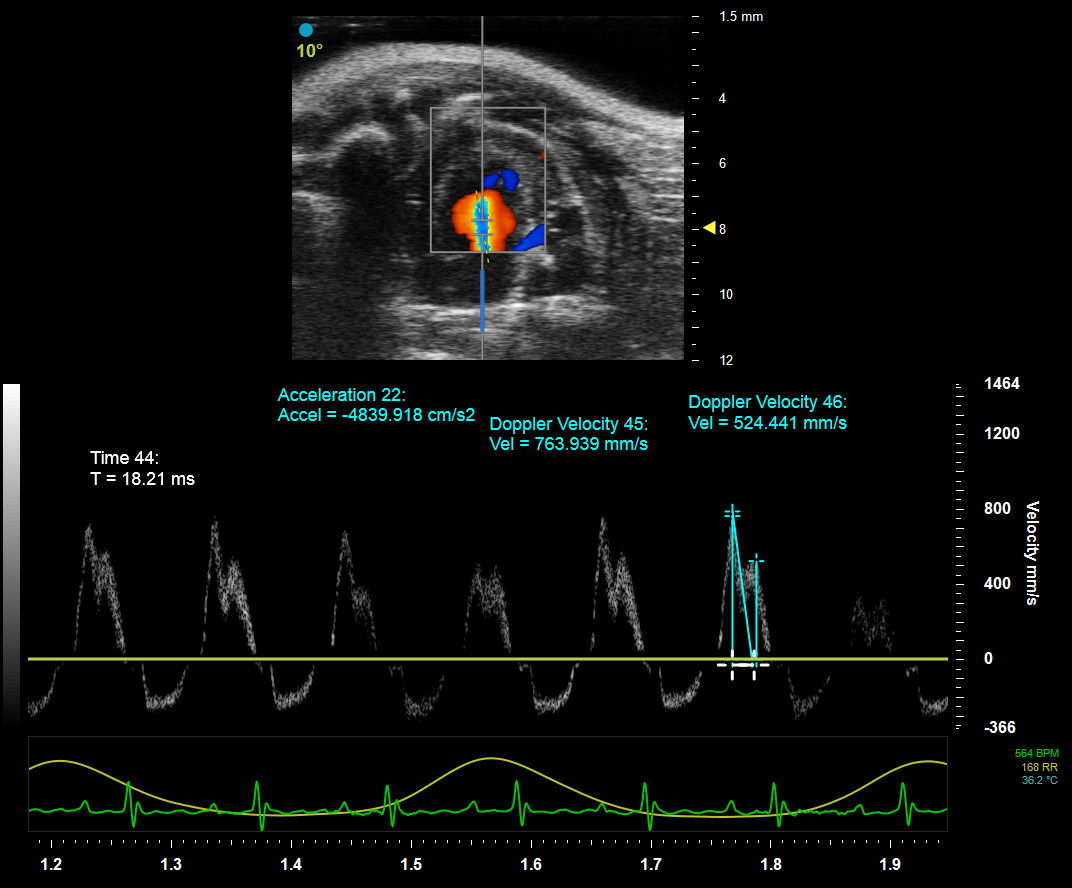

Supplement: Supplementary file 7 — Figure 2A Echo raw data part 2 [file 44321_2025_227_MOESM7_ESM.zip › Fig2 Echo pt 2/2-2 4w base/20220629091722400/20220629092251866.png]

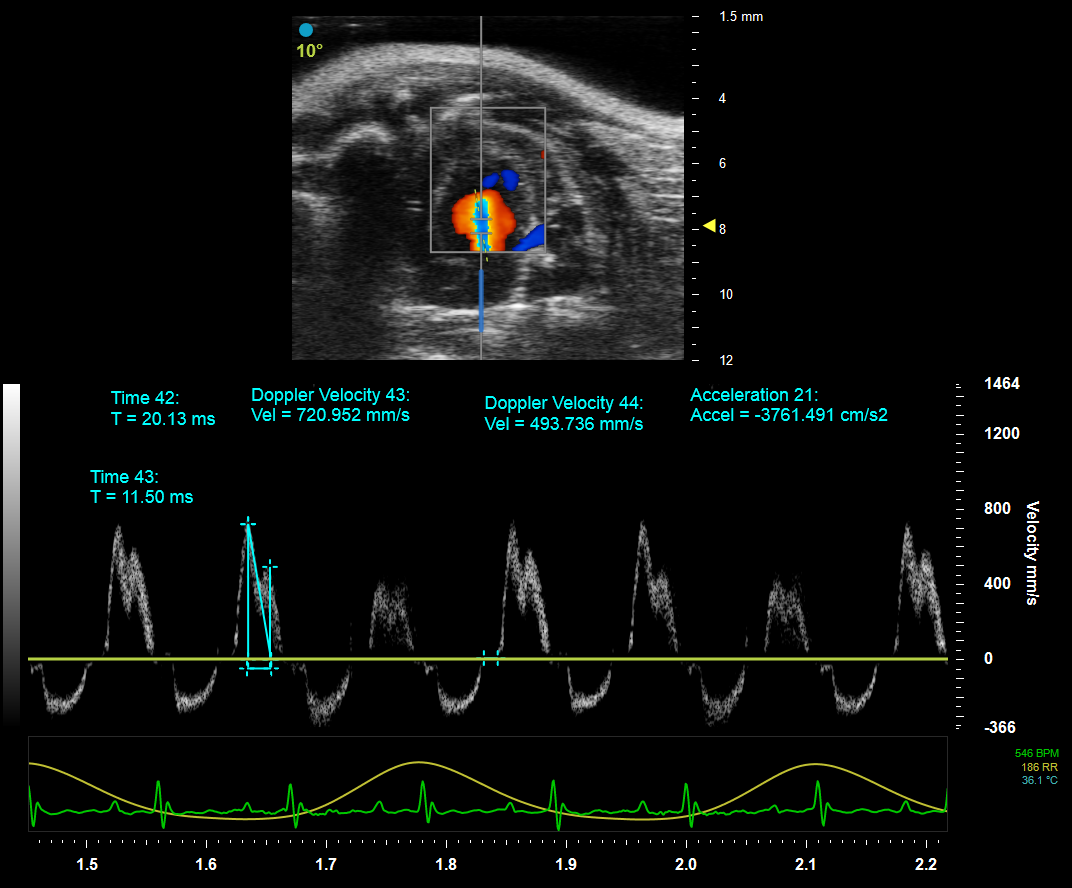

Supplement: Supplementary file 7 — Figure 2A Echo raw data part 2 [file 44321_2025_227_MOESM7_ESM.zip › Fig2 Echo pt 2/2-2 4w base/20220629091722400/20220629092303336.png]

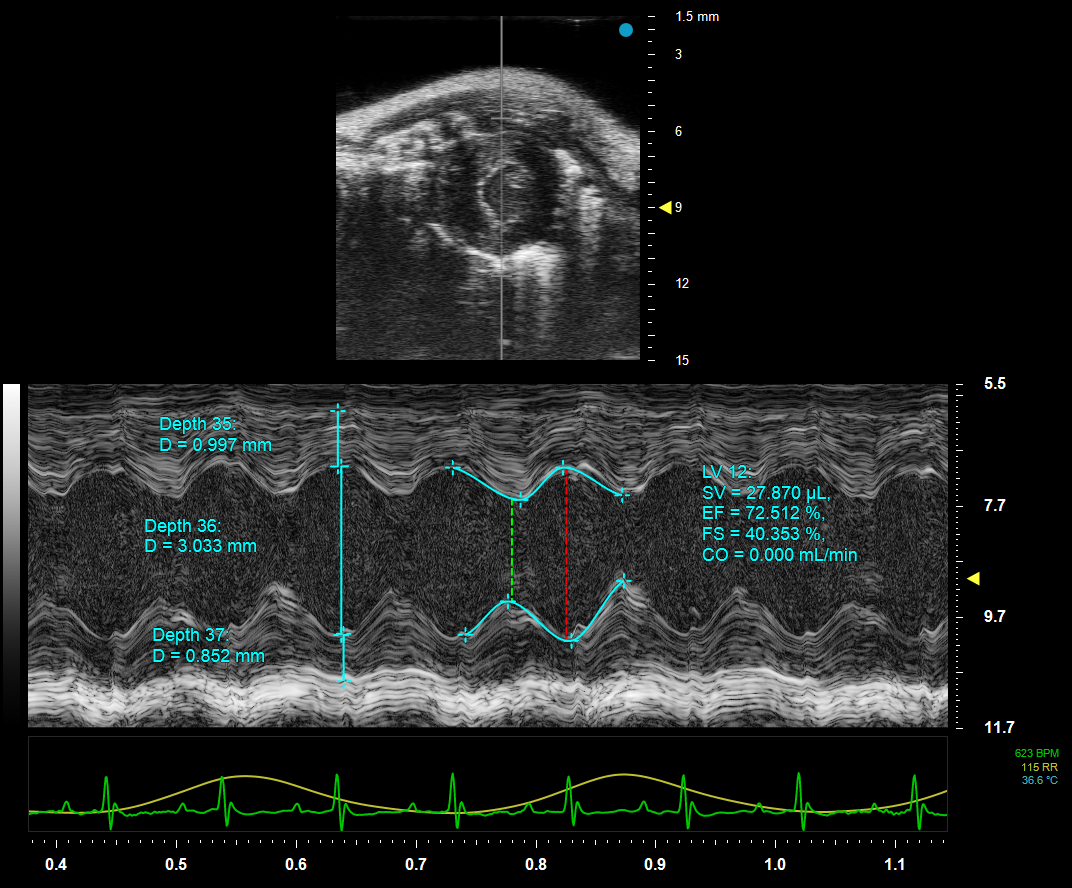

Supplement: Supplementary file 7 — Figure 2A Echo raw data part 2 [file 44321_2025_227_MOESM7_ESM.zip › Fig2 Echo pt 2/2-2 4w base/20220629091722400/20220629093403724.png]

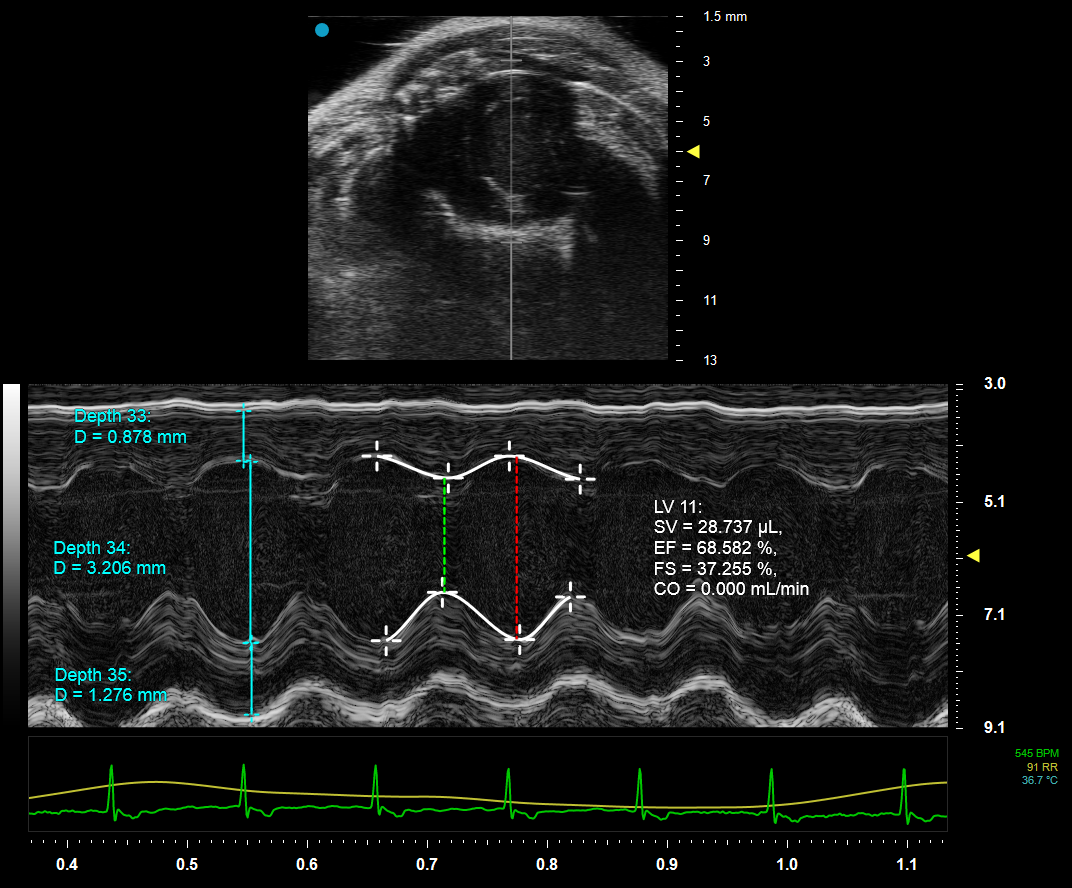

Supplement: Supplementary file 7 — Figure 2A Echo raw data part 2 [file 44321_2025_227_MOESM7_ESM.zip › Fig2 Echo pt 2/2-3 6w/20220711153116410/20220711153948456.png]

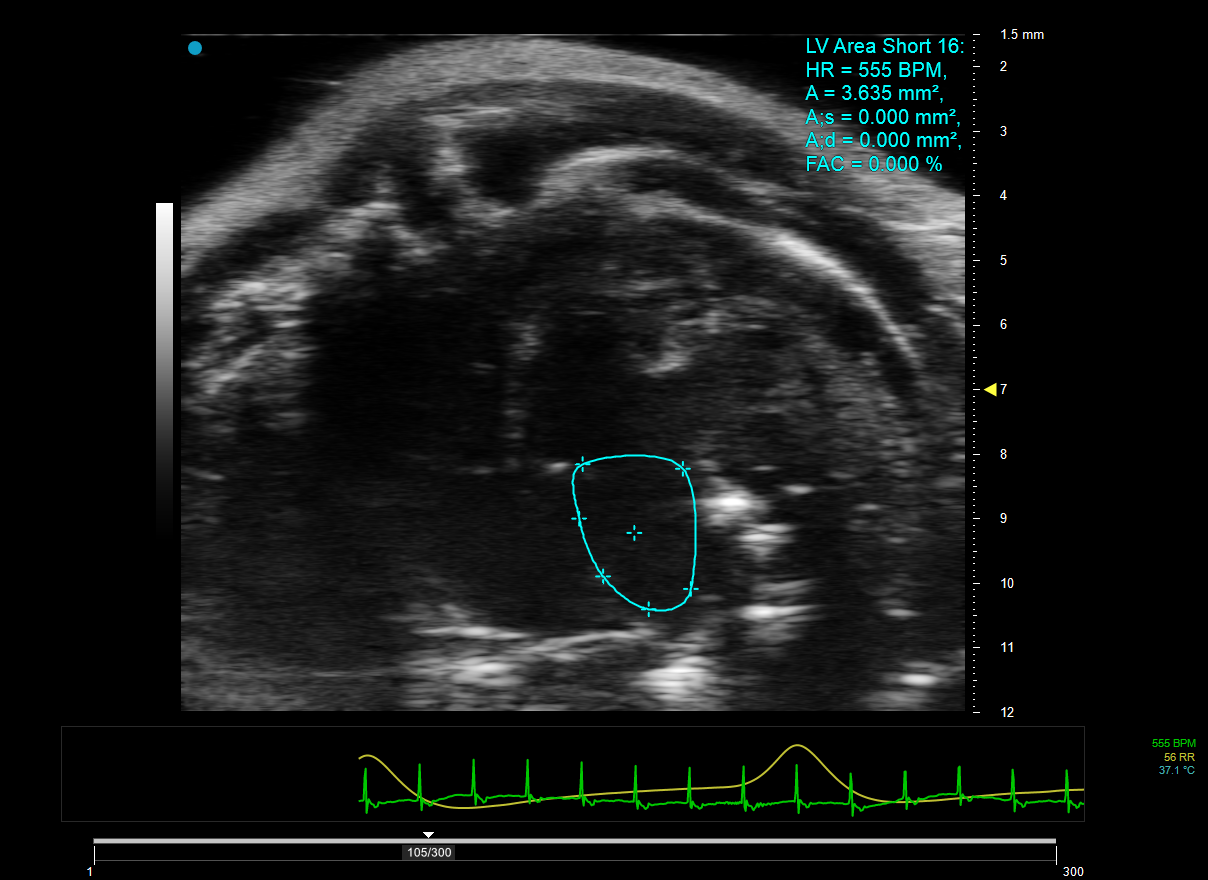

Supplement: Supplementary file 7 — Figure 2A Echo raw data part 2 [file 44321_2025_227_MOESM7_ESM.zip › Fig2 Echo pt 2/2-3 6w/20220711153116410/20220711154447340.png]

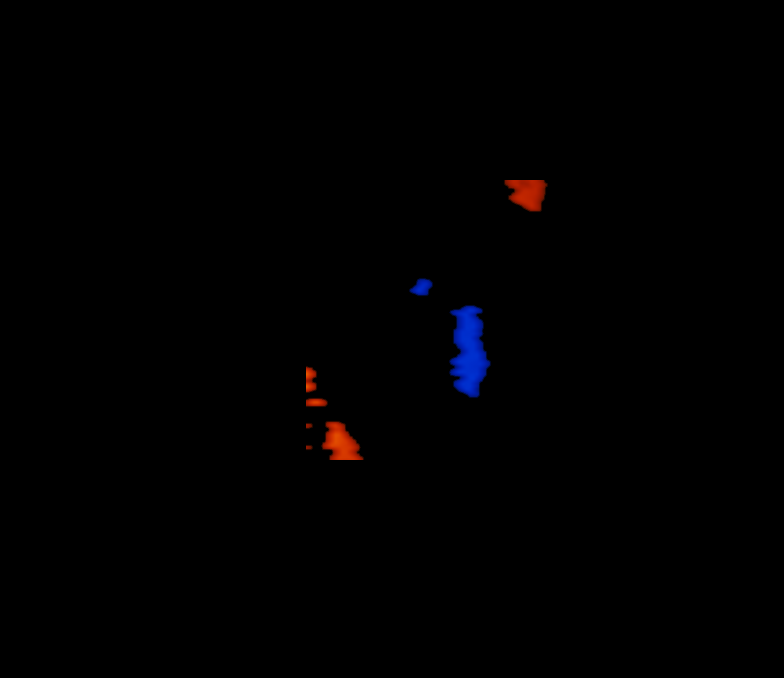

Supplement: Supplementary file 7 — Figure 2A Echo raw data part 2 [file 44321_2025_227_MOESM7_ESM.zip › Fig2 Echo pt 2/2-3 6w/20220711153116410/20220711154919933.overlay.bmp]

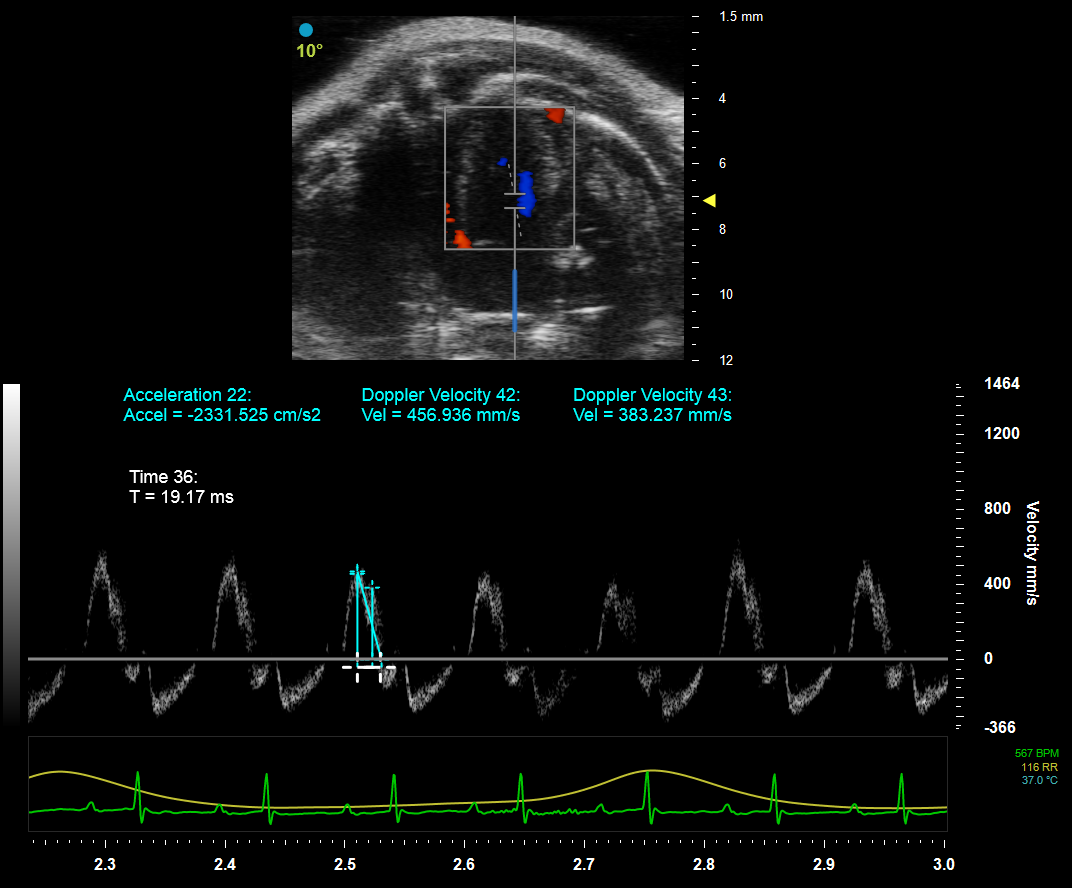

Supplement: Supplementary file 7 — Figure 2A Echo raw data part 2 [file 44321_2025_227_MOESM7_ESM.zip › Fig2 Echo pt 2/2-3 6w/20220711153116410/20220711154919933.png]

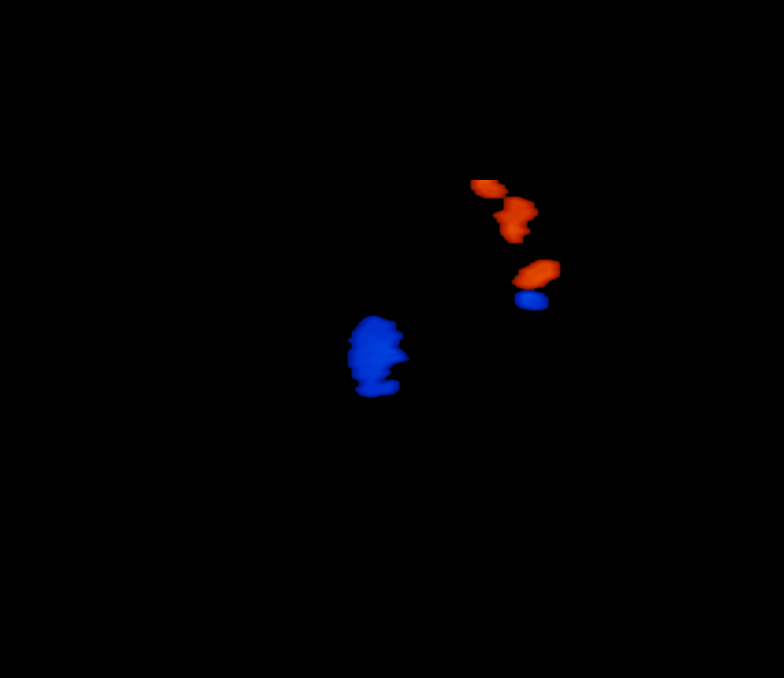

Supplement: Supplementary file 7 — Figure 2A Echo raw data part 2 [file 44321_2025_227_MOESM7_ESM.zip › Fig2 Echo pt 2/2-3 6w/20220711153116410/20220711154932735.overlay.bmp]

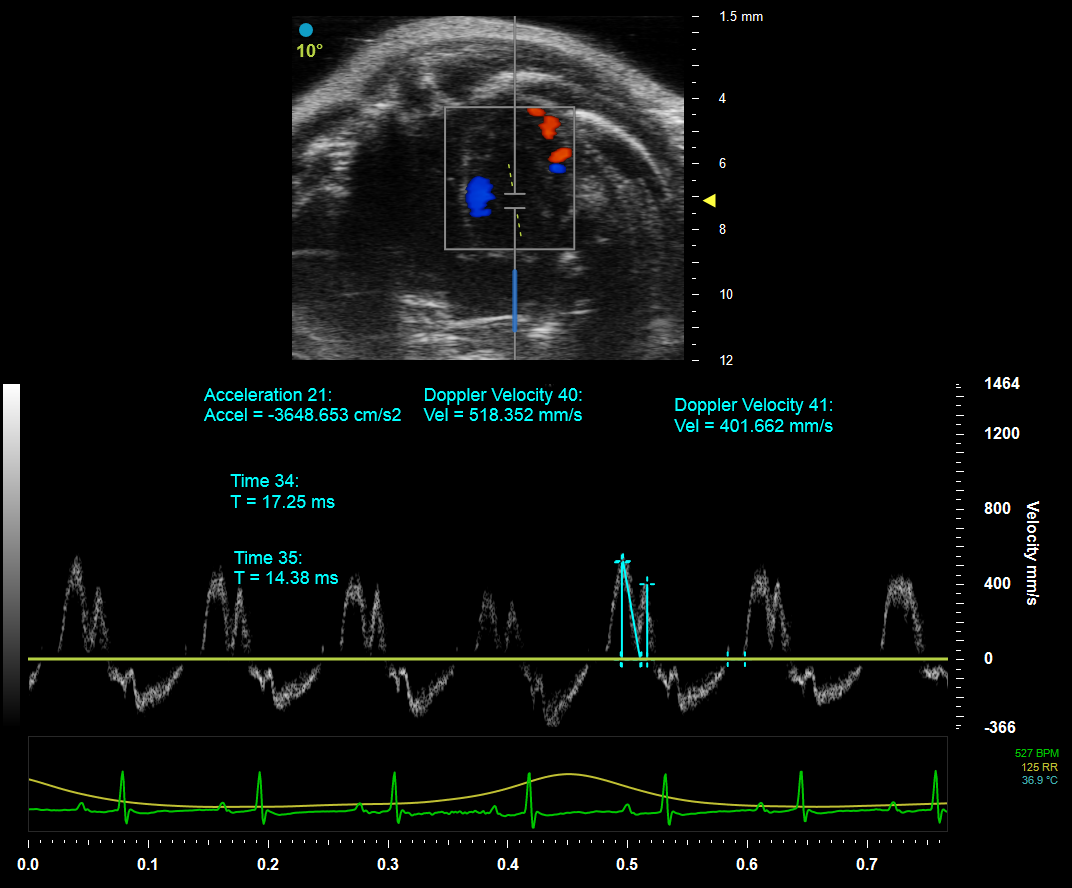

Supplement: Supplementary file 7 — Figure 2A Echo raw data part 2 [file 44321_2025_227_MOESM7_ESM.zip › Fig2 Echo pt 2/2-3 6w/20220711153116410/20220711154932735.png]
