# Supplementary material for: Formyl-peptide receptor type 2 activation mitigates heart and lung damage in inflammatory arthritis
Source: EMBO Mol Med. 2025 Apr 3;17(5):1153–83. doi: 10.1038/s44321-025-00227-1 (PMC12081931; doi:10.1038/s44321-025-00227-1)
Supplement: Supplementary file 14 — Expanded View Figures [file 44321_2025_227_MOESM14_ESM.pdf]

Expanded View Figures

A

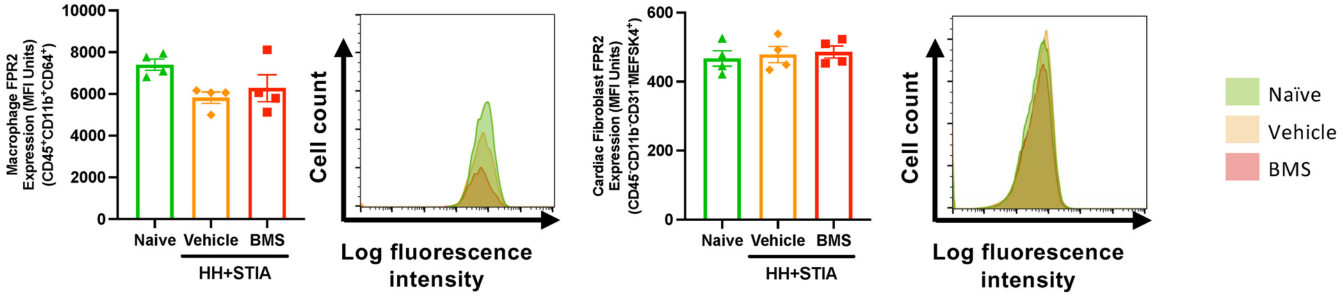

	Naïve Control		HH+STIA Vehicle		HH+STIA BMS	
	MFI Units (a.u.)	Frequency (%)	MFI Units (a.u.)	Frequency (%)	MFI Units	Frequency (%)
Myeloid Cells	6939 ± 258	60 ± 2	4679 ± 192	3 ± 1	5475 ± 813	45 ± 6
Macrophages	7399 ± 269	60 ± 2	5820 ± 276	48 ± 3	6276 ± 647	50 ± 5
Neutrophils	621 ± 40	42 ± 3	467 ± 45	28 ± 3	467 ± 44	30 ± 5
Fibroblasts	467 ± 22	2 ± 0.1	478 ± 23	2 ± 0.2	486 ± 18	2 ± 0.1
Endothelial Cells	271 ± 24	5 ± 0.5	280 ± 27	6 ± 0.7	258 ± 12	5 ± 0.4

B

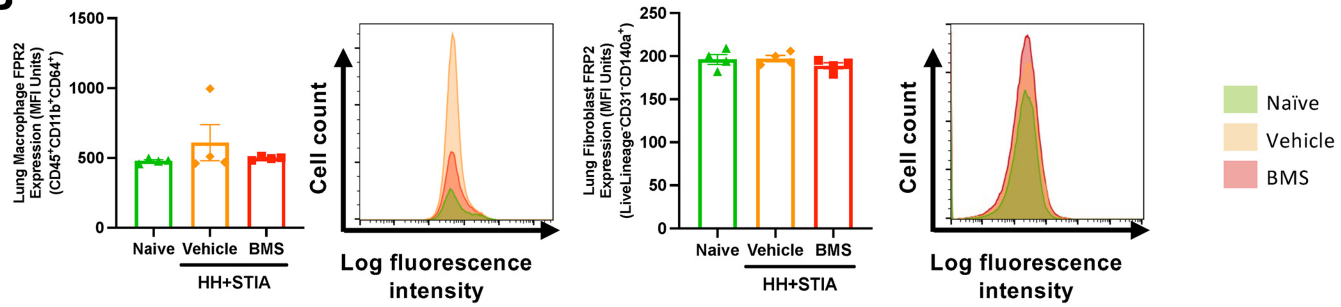

	Naïve Control		HH+STIA Vehicle		HH+STIA BMS	
	MFI Units (a.u.)	Frequency (%)	MFI Units (a.u.)	Frequency (%)	MFI Units (a.u.)	Frequency (%)
Myeloid Cells	374 ± 11	5 ± 1	534 ± 109	4 ± 1	437 ± 19	3 ± 0.5
Macrophages	479 ± 9	12 ± 1	609 ± 129	13 ± 6	498 ± 6	8 ± 0.9
Neutrophils	576 ± 11	48 ± 2	636 ± 90	54 ± 12	559 ± 36	45 ± 6
Fibroblasts	196 ± 6	2 ± 0.1	197 ± 4	2 ± 0.2	189 ± 3	2 ± 0.5
Endothelial Cells	189 ± 5	3 ± 0.1	173 ± 6	3 ± 0.3	166 ± 8	3 ± 0.6

Figure EV1. Formyl peptide receptor 2 (FPR2) expression in cardiac and lung cells.

HH + STIA was induced as in Fig. 1. From week 4, mice were treated with either vehicle (100 µl per os daily) or BMS235 (3 mg/kg per os daily). At the end of week 6, hearts and lungs were harvested, digested and processed for flow cytometry analysis. (**A**) Top panels, representative histograms for FPR2 expression on cardiac macrophages and fibroblasts. Table, summary data for the reported cardiac cell types. (**B**) Top panels, representative histograms for FPR2 expression on lung macrophages and fibroblasts. Table, summary data for the reported lung parenchyma cell types. Cardiac cell populations were defined by the following markers: myeloid cells, CD45+CD11b+; macrophages, CD45+CD11b+CD64+; neutrophils, CD45+CD11b+Ly6G+; endothelial cells, CD45-CD11b-CD31+; fibroblasts, CD45-CD11b-CD31-MEFSK4+. Lung cell populations were defined by the following markers: myeloid cells, CD45+CD11b+; macrophages, CD45+CD11b+CD64+; neutrophils, CD45+CD11b+Ly6G+; lung endothelial cells, Lineage-CD31+; fibroblasts, Lineage-CD31-CD140a+. Data are mean ± SEM of $n = 4$ (naive), $n = 4$ (HH + STIA+Vehicle) and $n = 4$ (HH + STIA + BMS) mice.

© The Author(s)

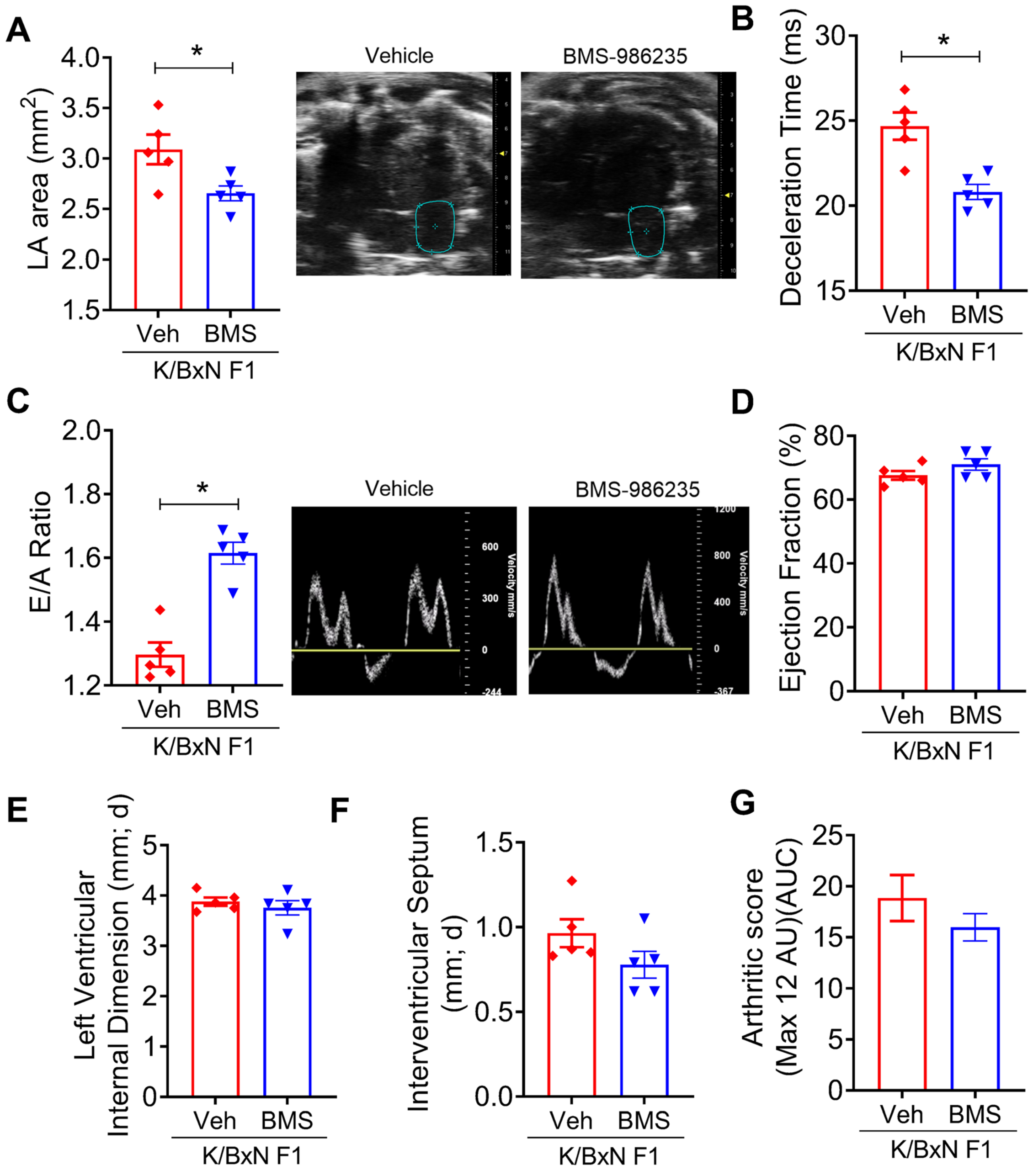

Figure EV2. Selective FPR2 agonism by BMS235 significantly attenuates cardiac diastolic dysfunction in K/BxN F1 mice.

K/BxN F1 mice were treated daily from week 4 to week 8 with vehicle or BMS235 (3 mg/kg p.o.). Echocardiography was performed at week 8 after 4 weeks of treatment. (**A**) Quantification of left atrial (LA) area (*, *P* value = 0.0029); Right-hand images: representative B-mode four-chamber echocardiograms and left atrial (light blue circles) area in K/BxN F1 mice. (**B**) Quantification of deceleration time (*, *P* value = 0.003). (**C**) Quantification of E/A ratio; Right-hand images: representative mitral flow patterns from pulsed-wave colour Doppler echocardiography (*, *P* value = 0.0003). (**D–G**) Quantification of four other cardiac parameters in the two experimental groups; (**D**) ejection fraction; (**E**) left ventricular dimension in diastolic (d) phase; (**F**) interventricular septum in diastolic (d) phase; (**G**) arthritic score, area under curve (AUC). Data are mean ± SEM of $n = 5$ mice per group. *$P < 0.05$ vs. vehicle group. One-way ANOVA with Tukey's multiple comparisons test. For (**B**, **J**): Kruskal–Wallis ANOVA followed by Dunn's multiple comparisons test.

 © The Author(s)

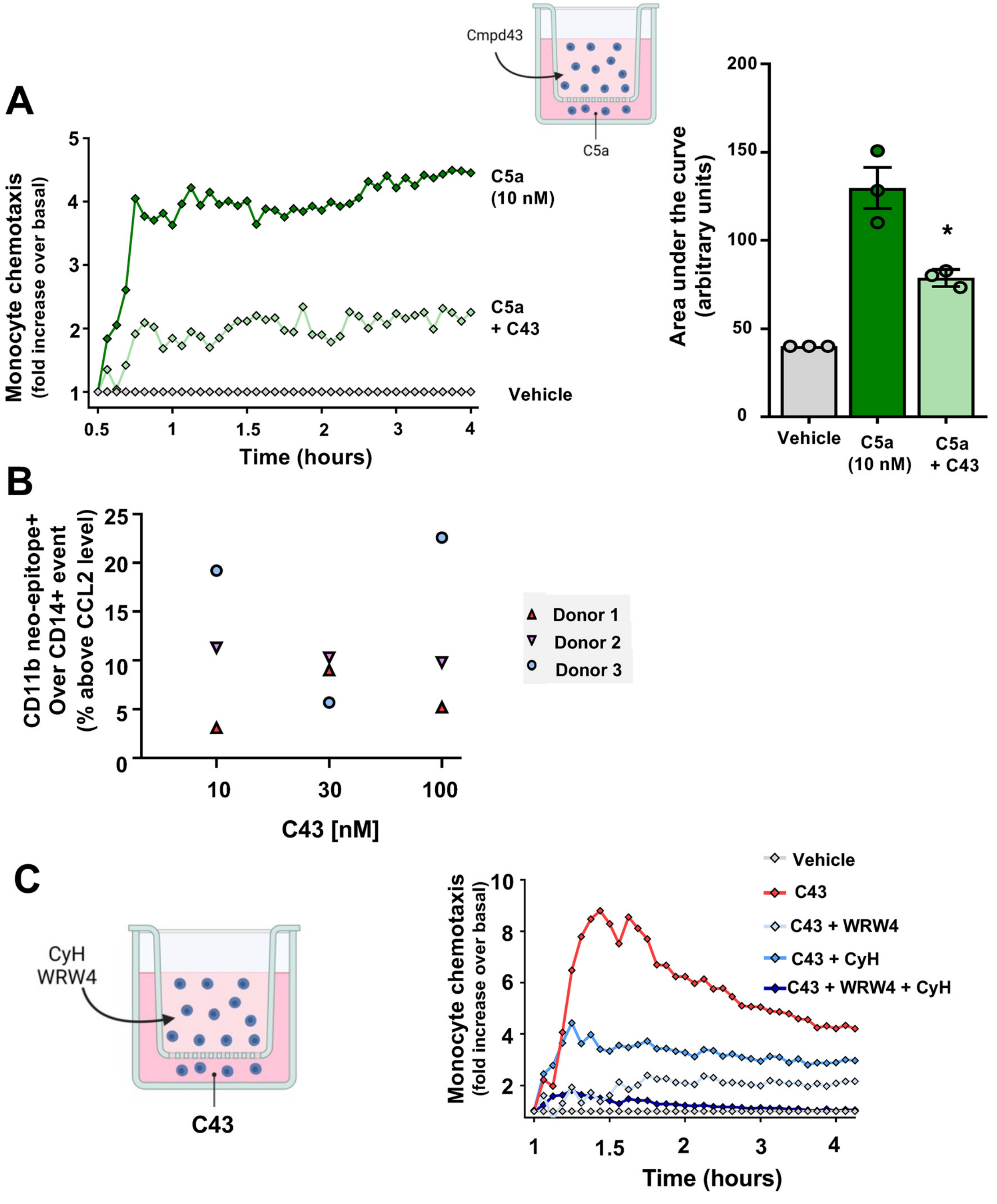

Figure EV3. Human monocyte reactivity experiments.

Chemotaxis of purified human peripheral blood monocytes was assessed using a xCELLigence™ DP system as in Fig. 8. (**A**) Inhibition of C5a (10 nM) induced monocyte chemotaxis by C43 (100 nM; 30 min pre-incubation); **P* value = 0.0051 vs. C5a data. (**B**) Additive effect of C43 to CCL2 (10 nM)-mediated neo-epitope expression. Data for each single donor (*n* = 3 in total) are presented. (**C**) C43 (100 nM)-mediated human monocyte chemotaxis; regulation by cyclosporin H (CyH, 1 µM) and WRW4 (1 µM) added to cells 30 min prior to beginning of the chemotaxis assay using the xCELLigence™ DP system. Quantitative data from multiple donor cells are given in the main text. For AUC-values: One-way ANOVA with Tukey's multiple comparisons test.

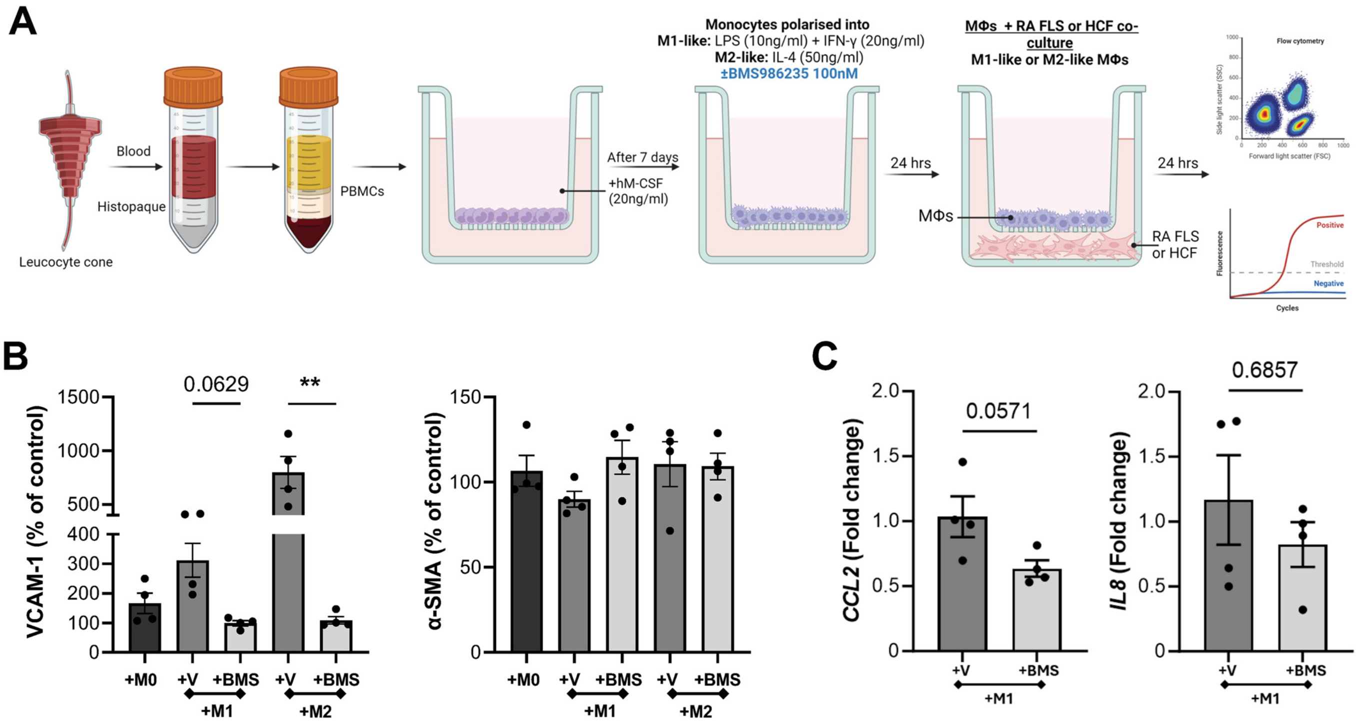

Figure EV4. Modulation of human macrophage/fibroblast crosstalk by BMS235.

(**A**) Schematic of the co-culture experiments with human monocyte-derived macrophages treated with vehicle or BMS235 (BMS, 100 nM) or vehicle control (0.1% DMSO) for 24 h prior to addition to either human RA synovial fibroblasts (FLS) or human cardiac fibroblasts (HCF). Fibroblast markers were quantified 24 h later. (**B**) VCAM-1 and α-SMA expression in human RA fibroblast-like synoviocytes (FLS). Data are mean ± SEM of 3–4 distinct cone preparations. (**, adjusted *P* value = 0.0025), one-way nonparametric Kruskal–Wallis, Dunn's multiple comparisons test. (**C**) Chemokine gene expression in human cardiac fibroblasts (HCF), where Ct values were normalised using 18S as a housekeeping gene and fold change was calculated relative to the geometric mean of the HCF co-cultured with vehicle control M1-like macrophages. Data are mean ± SEM of 4 distinct macrophage preparations. Statistical analysis is nonparametric *t*-test Mann-Whitney test.

 © The Author(s)

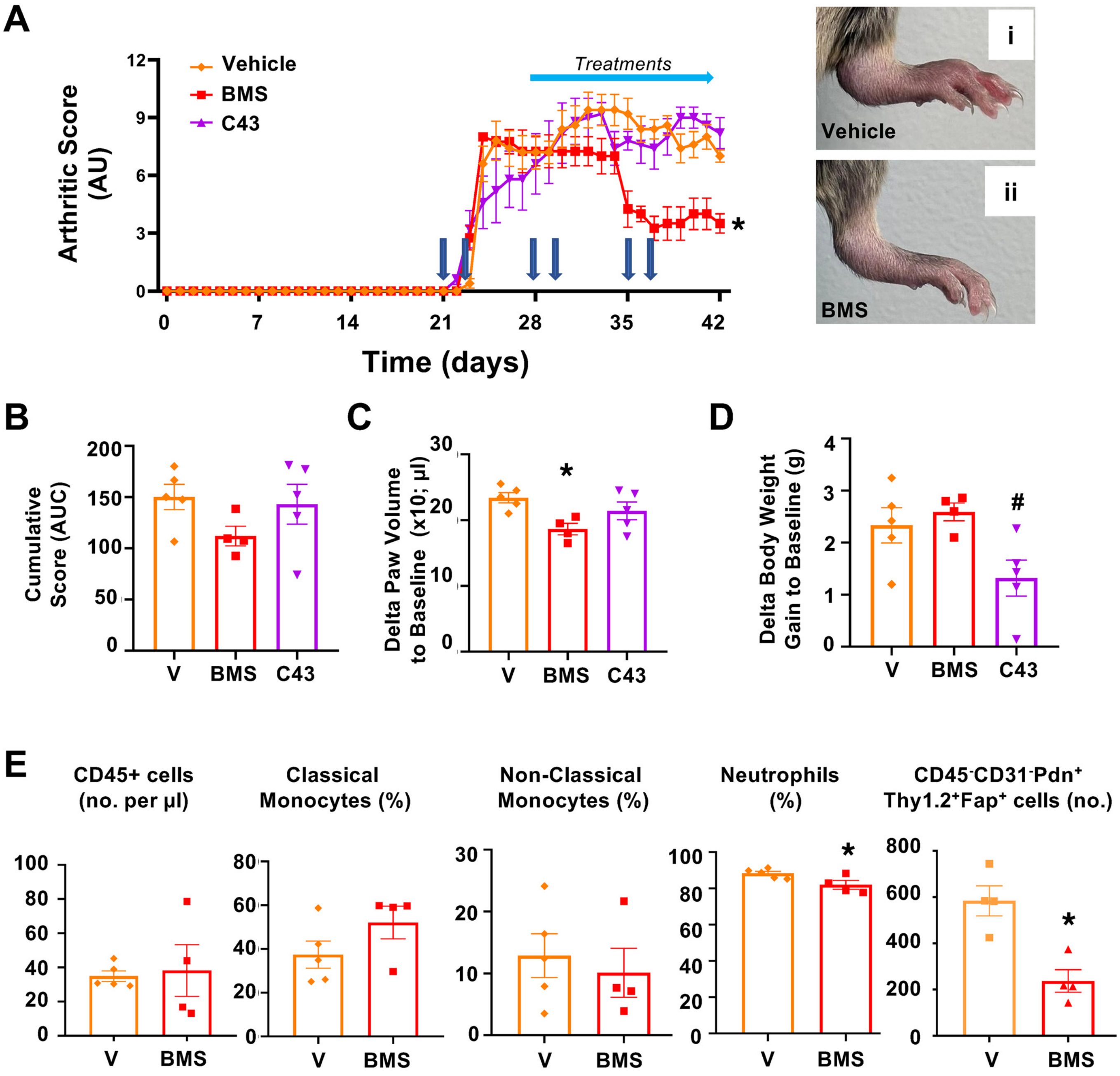

Figure EV5. Impact of FPR2 agonism on joint disease.

Figure EV5. Impact of FPR2 agonism on joint disease. HH + STIA was obtained as in Fig. 1. From week 4, mice were treated with either vehicle or BMS235 (3 mg/kg per os) or C43 (10 mg/kg per os) daily. (**A**) Time course of the arthritic score (arrows indicate serum injections; bar, treatment time). Representative images of arthritic score: i) score ≥9, ii) score ~6. (**B**) Cumulative value for the arthritic score. Area under the curve (AUC). (**C**) Oedema shown as delta paw volume between day 0 and day 42 (*, adjusted *P* value = 0.0085). (**D**) Change in body weight between day 0 and day 42 (#, adjusted *P* value = 0.0155). (**E**) Cellular characterisation from paws collected at day 42. Classical monocytes: CD45+CD11b+CD115+CD43-Ly6Chigh; non-classical monocytes: CD45+CD11b+CD115+CD43+Ly6Clow. Neutrophils: CD45+CD11b+CD115-Ly6G+ (*P* value = 0.0398). Proinflammatory fibroblasts: Thy1.2+CD45-CD31-Pdpn+Fap+ (*P* value = 0.0053). Data are mean ± SEM of $n = 4–5$ mice per group. *$P < 0.05$ vs. vehicle; #$P < 0.05$ vs. BMS group. For (**A**): Two-way ANOVA with Tukey's multiple comparisons test. For (**B–D**): one-way ANOVA with Tukey's multiple comparisons test. For (**E**): unpaired two-tailed *t*-test.

© The Author(s)
